# InvitroSPI and a large database of proteasome-generated spliced and non-spliced peptides

**DOI:** 10.1038/s41597-022-01890-6

**Published:** 2023-01-10

**Authors:** Hanna P. Roetschke, Guillermo Rodriguez-Hernandez, John A. Cormican, Xiaoping Yang, Steven Lynham, Michele Mishto, Juliane Liepe

**Affiliations:** 1 Max-Planck-Institute for Multidisciplinary Sciences (MPI-NAT), 37077 Göttingen, Germany; 2 Centre for Inflammation Biology and Cancer Immunology (CIBCI) & Peter Gorer Department of Immunobiology, King’s College London (KCL), SE1 1UL London, UK; 3 Francis Crick Institute, NW1 1AT London, UK; 4 Proteomics Core Facility, James Black Centre, King’s College London (KCL), SE5 9NU London, UK

**Keywords:** Proteasome, Antigen processing and presentation

## Abstract

Noncanonical epitopes presented by Human Leucocyte Antigen class I (HLA-I) complexes to CD8^+^ T cells attracted the spotlight in the research of novel immunotherapies against cancer, infection and autoimmunity. Proteasomes, which are the main producers of HLA-I-bound antigenic peptides, can catalyze both peptide hydrolysis and peptide splicing. The prediction of proteasome-generated spliced peptides is an objective that still requires a reliable (and large) database of non-spliced and spliced peptides produced by these proteases. Here, we present an extended database of proteasome-generated spliced and non-spliced peptides, which was obtained by analyzing *in vitro* digestions of 80 unique synthetic polypeptide substrates, measured by different mass spectrometers. Peptides were identified through invitroSPI method, which was validated through *in silico* and *in vitro* strategies. The peptide product database contains 16,631 unique peptide products (5,493 non-spliced, 6,453 *cis*-spliced and 4,685 *trans*-spliced peptide products), and a substrate sequence variety that is a valuable source for predictors of proteasome-catalyzed peptide hydrolysis and splicing. Potential artefacts and skewed results due to different identification and analysis strategies are discussed.

## Background & Summary

**Fig. 1:**
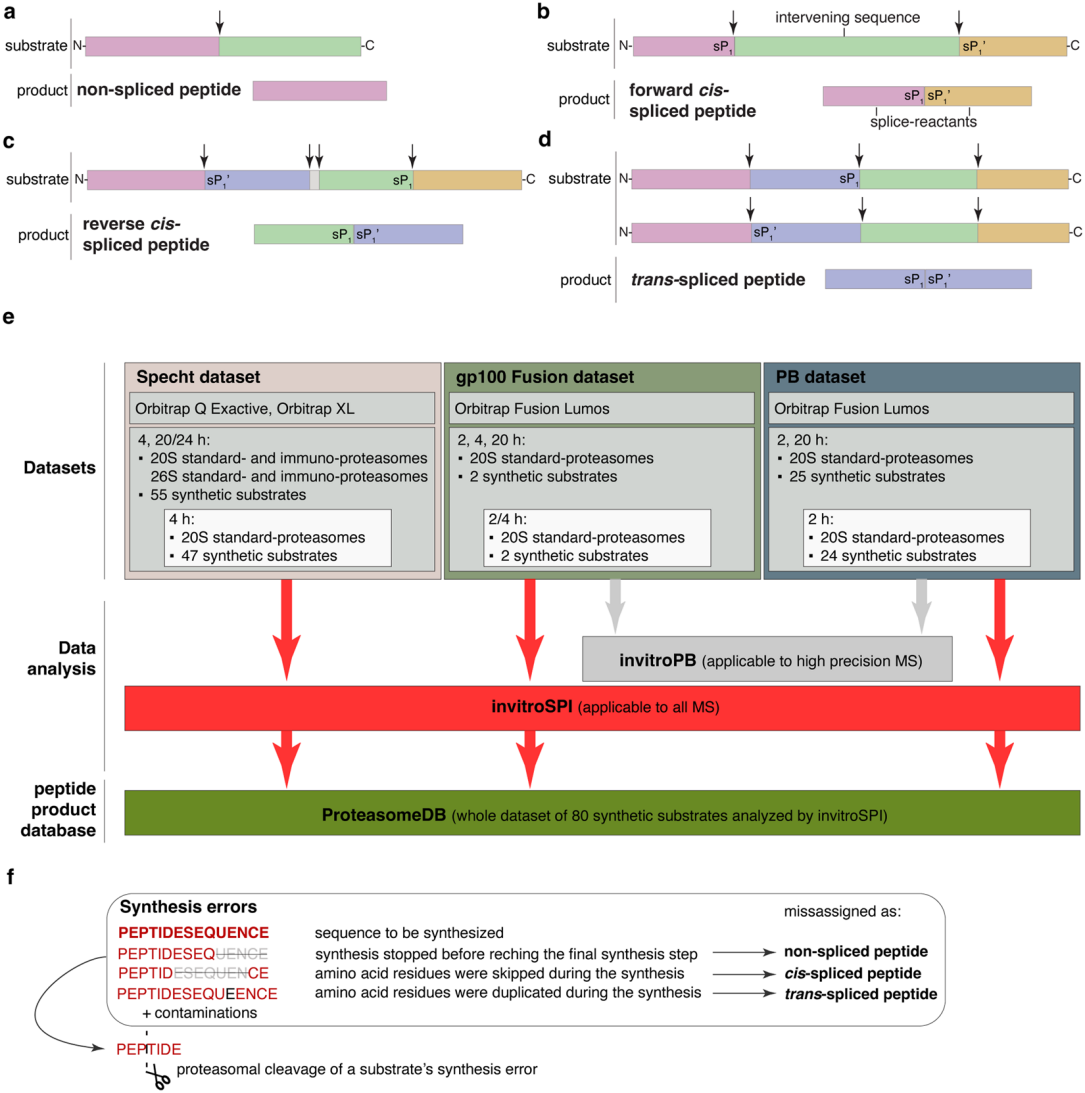
Proteasome-generated non-spliced and spliced peptides, and overview of method and dataset application. Proteasomes form: (**a**) non-spliced peptides via peptide hydrolysis, (**b-d**) spliced peptides through ligation of two non-contiguous splice-reactants either derived from the same protein molecule (*cis*-spliced peptides, **b, c**) or from two distinct molecules of the same protein or two distinct proteins (*trans*-spliced peptides, **d**). In **b-c**, peptide fragment ligation can occur in forward order, *i.e*., following the orientation from N- to C-terminus of the parental protein (forward *cis*-peptide splicing; **b**), or in reverse order (reverse *cis*-peptide splicing; **c**). The two ligated fragments are named splice-reactants, and their junction is named splice-site. The C-terminus of the first (N-terminal) splice-reactant is named sP_1_, whilst the N-terminus of the second (C-terminal) splice-reactant is named sP_1_’. The sequence segment between two splice-reactants is called the intervening sequence. Arrows represent the substrate cleavage sites used by proteasome catalytic Thr1. (**e**) Overview of methods and datasets described in this study. (**f**) Substrate synthesis errors. Various forms of synthesis errors could result in alleged non-spliced and/or spliced peptides. Those synthesis errors are captured using control measurements. Furthermore, alleged spliced synthesis errors can be trimmed by the proteasome. All such spliced peptides of which a precursor is identified in control measurements are removed by invitroSPI but not by invitroPB.

Despite being well known as proteolytic enzymes for four decades, the ability of proteasomes to catalyze the reverse reaction – namely, proteasome-catalyzed peptide splicing (PCPS) – was only identified in 2004, when two independent groups identified the first examples of tumor-associated spliced epitopes^1,2^. The proteolytic activity of these proteases, which is mediated by peptide hydrolysis (Fig. 1a), has been investigated from many angles and in many experimental and translational settings. Indeed, proteasomes degrade most of the cytoplasmic proteins - including transcription factors, obsolete, damaged or wrongly transcribed proteins - and changes in their proteolytic activity have been associated with many pathological conditions. Much less is known about PCPS, which comprises the ligation of two non-contiguous peptide fragments (*i.e*., splice-reactants) of the same molecule (*cis*-spliced peptides; Fig. 1b,c) or from two distinct molecules (*trans*-spliced peptides; Fig. 1d)^3^. Although *trans*-spliced peptides have been identified in both *in vitro* experiments with purified proteasomes^4–8^, *in cellula*^9^, and in HLA-I immunopeptidomes - *i.e*., in the pool of peptides bound to HLA-I complexes^10^ - their immunological relevance is still an enigma. In contrast, the immunological relevance of *cis*-spliced peptides has been evident since their first identification and has likely been a major driver for the development of methods for their identification. From few pioneering studies we know that many *cis*-spliced peptides are produced by proteasomes and presented by HLA-I molecules of various cells^10–14^. They can target CD8^+^ T cell responses against otherwise neglected bacterial antigens *in vivo*, in a mouse model of *Listeria monocytogenes* infection^15^. They can activate CD8^+^ T cells specific for *Listeria monocytogenes* or Human Immunodeficiency virus (HIV) through cross-recognition *ex vivo*^14,16^. Preliminary *in silico* studies suggest that *cis*-spliced peptides may not play an immunologically significant role in CD8^+^ T cell tolerance, although potential cases of viral-human epitope mimicry associated with autoimmune diseases cannot be excluded^17,18^. *Cis*-spliced peptides can carry cancer-specific mutations^6,19^, and are recognized by CD8^+^ T cells in peripheral blood of melanoma patients^11,20^ and healthy donors^20,21^. A melanoma patient with metastasis was cured through adoptive T cell therapy using an autologous tumor-infiltrating lymphocyte clone, which was proved, in a later study, to be specific for a *cis*-spliced epitope derived from a melanoma-associated antigen^22,23^.

The location of the catalytic sites within the inner chamber of the proteasome barrel can be one of the reasons for efficient PCPS activity^24^, although proteases with different structures can catalyze peptide splicing as well^25–28^.

Both peptide hydrolysis and peptide splicing can be catalyzed by different proteasome isoforms, such as 20 S standard-, immuno-, and thymo-proteasomes, as well as by 20 S proteasomes coupled to regulatory subunits, such as 26 S proteasomes^3,5,7,8,29,30^. Both catalytic reactions seem to be highly tuned mechanisms, wherein the residues surrounding the substrate cleavage- and splice-sites, as well as catalytic dynamics, may play a pivotal role^4,5,25,31–34^. This implies that, by dissecting these driving factors, we may predict which spliced and non-spliced peptides are produced by proteasomes. PCPS predictors may be integrated in some of the pipelines that have been proposed for targeted epitope discovery and immunotherapies^6,15,35–41^.

Such PCPS predictors should be trained on robust, validated databases of non-spliced and spliced peptides produced by proteasomes. These databases should be large and diverse enough to ensure the generalizability of the obtained predictions.

The identification of spliced peptides in HLA-I immunopeptidomes has a number of technical hurdles. This has ignited an intense controversy and the proliferation of identification methods with discordant performance, and thereby divergent estimation of spliced peptide frequency in HLA-I immunopeptidomes (for more details see^24,42–44^). Theoretically, these technical hurdles are less pronounced in a controlled experimental set up, such as *in vitro* digestion of synthetic polypeptides by purified proteasomes, measured by mass spectrometry (MS). Indeed, this kind of assay requires a much smaller spliced peptide reference database and hence results in a significantly smaller theoretical search space in the MS data analysis compared to HLA-I immunopeptidome analysis. Correspondence between *in vitro* experiments carried out with purified 20 S proteasomes and *in cellula* and *in vivo* experiments has been demonstrated in various studies investigating both viral and tumor epitopes^2,15,20,23,29,45–53^. 20 S proteasomes can degrade intrinsically disordered proteins *in vitro* and *in cellula*^54–56^. Recently, Specht *et al*.^8^ and Paes *et al*.^57^ published the first two datasets of *in vitro* digested synthetic polypeptides, and systematically identified non-spliced and spliced peptides produced by proteasomes through the analysis of MS measurements by methods specifically developed for this purpose. Our study^8^ investigated the degradation of 55 synthetic polypeptide substrates (‘Specht dataset’), whereas the dataset published by Paes *et al*.^57^ contained 25 substrates (‘ PB dataset’). Despite the attempts, both datasets were too small for a statistically robust analysis of the sequence motifs (see Technical Validation Section), which we suggest being the cornerstone of any PCPS predictor development. The two studies applied different methods for the identification of non-spliced and spliced peptides. The outcomes, in terms of spliced peptide frequency and features, diverged, thereby rendering unwise the merging of the two databases of non-spliced and spliced peptides produced by proteasomes. Indeed, since the objective of our study was the generation of a database of non-spliced and spliced peptides produced by proteasomes through the degradation of 80 synthetic polypeptides, all digestions should be analyzed with a single peptide identification method to avoid biases arising from differences in the respective identification algorithms. Therefore, we developed an improved version of our method – namely, *in vitro* Spliced Peptide Identifier (invitroSPI; Fig. S1) – and implemented Paes’ method (referred to as invitroPB method; Fig. S2); then, we applied both of them to a new small dataset (namely, ‘gp100 Fusion dataset’) and then to the larger PB dataset, and compared their outcome by using state-of-the-art methods for the evaluation of MS2 spectra and other MS features (Fig. 1e). Based on the latter outcomes, we then applied invitroSPI to the whole dataset containing the Specht dataset, the PB dataset, and the new gp100 Fusion dataset. Thereby, we generated a database of non-spliced (n = 5,493), *cis*-spliced (n = 6,453) and *trans*-spliced (n = 4,685) peptides (ProteasomeDB) - produced by proteasomes, derived from 80 synthetic polypeptide substrates and analyzed through the same method - which may be informative enough for PCPS predictor development.

## Methods

### Statistical analysis

All statistical tests were done in R. Differences in distributions have been tested using either a two-sided Student’s t-test, a two-samples Wilcoxon test or a Kolmogorov-Smirnov test, depending on the data distribution. Bootstrapping was applied by sampling 80% of the data repeatedly (n = 200 iterations) and calculating the 90% confidence interval over all bootstrap results.

### Peptide synthesis and proteasome purification

All synthetic peptides used for MS2 spectrum comparison were synthesized using Fmoc solid phase chemistry. The 20 S standard proteasomes used in this study were purified from K562 cell line, as described elsewhere^8^. Proteasome concentration was measured by Bradford staining and verified by Coomassie staining of an SDS-Page gel, as shown elsewhere^58^. The purity of the proteasome preparation using this protocol has previously been shown^30^. The Specht dataset was generated using human 20 S and 26 S standard- and immuno-proteasomes^8^, the PB dataset was produced using human 20 S standard proteasomes^57^, and the gp100 Fusion dataset was produced using human 20 S standard proteasomes (Fig. 1e).

### *In vitro* digestions and MS measurements

As part of the gp100 Fusion dataset, the synthetic polypeptides TSN2 [VSRQLRTKAWNRQLYPEWTEAQR] and TSN89 [RTKAWNRQLYPEW] (final concentration of 40 μM) were digested for different time points (0, 2, 4, 20 h) at 37 °C by either 0.75 μg (TSN2) or 1.5 μg (TSN89) 20 S proteasomes in 40 μl TKMD buffer (50 mM Tris/HCl-pH 7.8, 20 mM KCl, 5 mM MgAc, 1 mM DTT). Reactions were stopped by acidification. *In vitro* digestions were measured through Orbitrap Fusion Lumos spectrometer at Centre of Excellence of MS (CEMS) at King’s College London (KCL) as follows: either 5 μl of *in vitro* digestion samples or 2 μl gp100-PMM_210325 synthetic peptide library were injected using an Ultimate 3,000 RSLC nano pump (both from ThermoFisherScientific). Briefly, peptides were loaded and separated by a nanoflow HPLC (RSLC Ultimate 3000) on an Easy-spray C18 nano column (50 cm length, 75 mm internal diameter; ThermoFisherScientific). Peptides were eluted with a linear gradient of 5%–55% buffer B (80% ACN, 0.1% formic acid) at a flow rate of 300 nl/min over 100 min at 45 °C. The instrument was programmed within Xcalibur 4.4 to acquire MS data using a “Universal” method by defining a 3 s cycle time between a full MS scan and MS2 fragmentation. We acquired one full-scan MS spectrum at a resolution of 120,000 at 200 m/z with a normalized automatic gain control (AGC) target (%) of 250 and a scan range of 300~1,600 m/z. The MS2 fragmentation was conducted using HCD collision energy (35%) with an orbitrap resolution of 30,000 at 200 m/z. The AGC target (%) was set up as 200 with a max injection time of 128 ms. A dynamic exclusion of 30 s and 1–7 included charged states were defined within this method.

Gp100-PMM_210325 synthetic peptide library contained peptides and splice-reactants previously identified (or just investigated) in TSN2 and TSN89 substrate degradations^5,20^. Each peptide was present in a concentration of 0.4 μM (Table S1).

The Specht^8^ and PB^57^ datasets were originally measured through either LTQ XL, Q Exactive Plus and Q Exactive Orbitrap or Fusion Lumos Orbitrap mass spectrometers, respectively.

All collected MS RAW files were converted to the Mascot Generic Format (MGF) using ProteoWizard msconvert, employing the vendor peak picking option. RAW files that contained XL Ion Trap and XL Orbitrap scans were split into separate files for each mass analyzer type. Afterwards, headers containing search parameters were added to the MGF files and matched using Mascot v2.7.01 and PEAKS v10.5 (and PEAKS v8.5) with a mass tolerance of either 10 ppm (for XL mass spectrometer), 6 ppm (for Q Exactive Orbitrap mass spectrometer) or 5 ppm (for Orbitrap Fusion Lumos mass spectrometer) on precursor masses. Mass tolerance of fragment ions was set at either 0.5 Da (for Iontrap XL mass spectrometer in CID mode), 20 ppm (for XL and Q Exactive Orbitrap mass spectrometers in HCD mode), 0.02 Da (for the Orbitrap Fusion Lumos mass spectrometer in HCD mode at Proteomics Core Facility, KCL), and 0.03 Da (for the Orbitrap Fusion Lumos mass spectrometer in HCD mode at Proteomics Core Facility, University of Oxford). All MS measurements derived from a given synthetic polypeptide substrate were analyzed together in all investigated methods.

### *In vitro* digestion datasets and peptide product database

In the Specht dataset (55 synthetic polypeptide substrates), *in vitro* digestions of 48 synthetic substrates have been measured by XL MS at Charité Shared Facility for MS, 4 and 10 synthetic substrates have been measured by Q Exactive Orbitrap at Charité Shared Facility for MS and by Q Exactive Orbitrap at MPI-NAT Core Facility for Proteomics, respectively. *In vitro* digestions of 47 synthetic substrates have been carried out with human 20 S standard proteasomes for 4 h. For four synthetic substrates, *in vitro* digestions have also been carried out with human 20 S immunoproteasomes. For one synthetic substrate, *in vitro* digestions have also been carried out with human 20 S and 26 S standard- and immuno-proteasomes^8^.

In the original PB dataset, *in vitro* digestions of 25 synthetic substrates have been measured by Orbitrap Fusion Lumos at the MS Centre of Jenner Institute (University of Oxford)^57^. To note, no product sequences were detected in the control PP9 (TSN108) substrate of the original PB dataset using Mascot search engine. Thus, potential synthesis errors and contaminants related to the TSN108 substrate could not be identified and removed in the final peptide product database (see invitroSPI and invitroPB method description below).

In the peptide product database published by Paes *et al*.^57^, *cis-*spliced peptides were detected in only 16 out of 25 synthetic substrates after 2 h digestion. After applying downstream filtering steps that were described by Paes *et al*.^57^, *i.e*., removing all peptides carrying the substrate’s N- or C-termini, the original peptide product database that contained *cis*-spliced peptides was restricted to 12 synthetic polypeptide substrates. This final peptide product database has been used for the latter part of the Technical Validation section (see below).

For the present study, we generated the gp100 Fusion dataset, which contained the gp100-derived TSN2 and TSN89 substrate digestions that have been measured through Orbitrap Fusion Lumos at Proteomics Core Facility (KCL). TSN2 and TSN89 substrates were already present in the Specht dataset, although the experiments were performed in different conditions, and were measured through a different mass spectrometer (Fig. 1e).

### Proteasome-generated peptide product database

Our whole peptide product database (ProteasomeDB) contains non-spliced and spliced peptides produced in proteasome-mediated *in vitro* digestions of 80 unique synthetic polypeptide substrates. The latter is the whole dataset containing the three datasets described above (Online-Table 1). The peptide products were identified by applying invitroSPI method. In the entire study, we reported the number of ‘unique peptides per substrate’, which we speculate will be more useful for the development of proteasome activity predictors than the ‘unique peptides’ unrelated to the substrate origin. Therefore, if a peptide sequence was generated, for example, from 2 distinct substrates, it was reported as two distinct unique peptides per substrate in this study. However, ProteasomeDB structure allows the user to adopt different strategies for the computation of unique peptides, depending on the user’s goal.

Peptides have been produced by various proteasome isoforms and conditions, in 0, 2, 4, 20/24 h *in vitro* experiments at 37 °C. Samples containing either only synthetic substrates – *i.e*., without proteasomes – left for 20 h at 37 °C, or synthetic substrates and proteasomes left for 0 h at 37 °C, have been used as negative control. For each substrate, 1–4 biological replicates have been carried out, and measured 1–5 times.

The length of synthetic polypeptide substrates varies from 13 to 47 amino acids (Online-Table 1). They have an amino acid frequency that is similar to the frequency present in the human proteome^8,57^. The polypeptides are derived from bacterial, viral and human proteins (largely antigens). In the Specht dataset (comprising 55 synthetic polypeptide substrates), there is a preponderant presence of tumor-associated or autoimmune disease-associated antigens. In the PB dataset (comprising 25 synthetic polypeptide substrates), there is a preponderant presence of HIV antigens. The species of origin of the substrate and unique identifier of the substrate sequences are attributes of our ProteasomeDB database (see Table 1).

**Table 1:** ProteasomeDB database description.

Column name	Description
sampleID	Unique identifier for every sample
sampleName	Sample Name used during experiment
filename	Mascot search result file name (available on PRIDE)
runID	Technical replicate number
protIsotype	Proteasome isoform used for digestion
digestTime	Elapsed digestion time (hours) at time of measurement
proteasomeSpecies	Species origin of used proteasomes
sampleDate	Sample date
instrument	Instrument used for measurement
fragmentation	Fragmentation method used for measurement
location	Measurement location
substrateSeq	Amino acid sequence of substrate
substrateOrigin	Protein origin of substrate
substrateSpecies	Species origin of substrate
substrateID	Unique identifier for a substrate sequence
pepSeq	Amino acid sequence of peptide products
scanNum	Scan number listed in the RAW file
rank	Peptide rank assigned by Mascot Server
ionScore	Ion score assigned by Mascot Server
qValue	q-value assigned by Mascot Server
productType	PCP: non-spliced peptide; PSP: spliced peptide
spliceType	cis: forward *cis-*spliced peptide; revCis: reverse *cis-*spliced peptide; trans: *trans-*spliced peptide; N/A: non-spliced peptide
positions	Location(s) of the peptide sequence in the synthetic polypeptide substrate
synErrSR2	Indication whether the C-terminal splice-reactant of a spliced peptide matches a non-spliced synthesis error; N/A: non-spliced peptide
charge	Ion charge
PTM	Post-translational modifications

Listed are the column names (attributes) in the ProteasomeDB database and their corresponding explanations.

Experiments have been carried out with synthetic polypeptides rather than the entire protein because purified proteasomes have been shown to hardly process entire proteins *in vitro*, likely because ligases and cofactors are lost during 20 S/26 S proteasome purification^59^. However, a correspondence between *in vitro* experiments - with synthetic polypeptides and purified proteasomes - and *in cellula* and *in vivo* experiments has been widely demonstrated (see text above).

Each digestion has been performed with a single polypeptide as substrate. Therefore, non-spliced and *cis*-spliced peptides could be produced by processing of a single molecule of the substrate (Fig. 1a–c), whereas *trans*-spliced peptides resulted from the ligation of two partially overlapping fragments derived from two molecules of the same substrate (Fig. 1d). *Trans*-spliced peptides with splice-reactants from two different substrate sequences were not possible because each *in vitro* digestion contained only one substrate rather than various substrates.

*In vitro* digestions have been performed at 0, 2, 4 and 20/24 h, and peptide products have been identified by applying invitroSPI method, which removed synthesis artefacts from the final list of identified peptide products (see Technical Validation section). To note, peptide synthesis artefacts can arise due to synthesis errors during the FMOC solid phase chemistry, peptides that contaminated the samples during their preparation, or other forms of contaminations. Both types of contaminations are termed synthesis errors, in this study. The synthesis errors generated during the peptide synthesis by FMOC solid phase chemistry could be: (i) truncated peptides that are shorter than the cognate synthetic polypeptide substrate at the N- and/or C-terminus, (ii) peptides lacking one or more residues within their sequence (*i.e*. not at the substrate termini), (iii) peptides containing the duplication of one (or more) amino acid. The example (i) could result in the wrong assignment of both non-spliced and spliced peptides, the example (ii) in the wrong assignment of *cis*-spliced peptides, and the example (iii) in the wrong assignment of *trans*-spliced peptides (Fig. 1f).

Since substrate degradation rates varied from substrate to substrate, from proteasome preparation to preparation, *in vitro* reaction conditions were set up to have the 2–4 h time points, wherein substrate molecules were still present in the reaction, and 20/24 h time point, wherein most of the substrate molecules have been processed by proteasomes. The presence of intact substrate molecules in the reaction can be determined by analyzing the MS RAW files linked to our database (see Data Record section).

Compared to the previous version of the peptide product database^8^, in ProteasomeDB, we expanded the number of substrates, their sequence variety and origin, as well as we strongly increased the number of digestion samples measured with high accuracy Orbitrap MS. In fact, ProteasomeDB contains proteasome-generated peptide products of 80 synthetic polypeptide substrates that have been measured with Orbitrap mass spectrometers with a mass tolerance of 5–6 ppm on precursor masses, and 20 ppm or 0.02–0.03 Da for fragment ions (Online-Table 1). Furthermore, the improved performance of invitroSPI increased the precision of peptide identification (see below).

ProteasomeDB is a CSV table, which contains 26 columns describing features of the identified peptides, the original substrate sequence, sample processing and instrument parameters (see Table 1 for a detailed description of the database columns/attributes). Additional to the information provided in the Specht database of peptide products^8^, this new database contains all possible multi-mapper peptides with their correct splice-type annotation (see Technical Validation section).

### Prediction of MS2 spectra

Prosit version 2020^60,61^ allows prediction of the MS2 spectra given a peptide sequence, precursor charge and calibrated collision energy. A predicted MS2 spectrum can be compared to the detected MS2 spectrum by computing a similarity score. In this study, we used the spectral angle between the L2 normalized spectra, also known as normalized spectral contrast angle^62^, which ranges from 0 (very bad match between MS2 spectra) to 1 (perfect match between MS2 spectra). The spectral angle consists of a transformation on the normalized dot product and corresponds to the loss metric on which Prosit was trained.

### Generation and analysis of simulated background databases

In order to identify proteasome specificities, a simulated background database containing a subset of all theoretically possible spliced and non-spliced peptides was generated, similar to what was previously described in Specht *et al*.^8^. The simulated background database reflected the peptide products that one would expect to be detected in absence of any proteasome specificities, *i.e*., under the assumption that each theoretically possible spliced peptide is generated with the same probability. The simulated background database was obtained by sampling uniformly a subset of all theoretically possible spliced and non-spliced peptides (*i.e*., a subset of the custom reference database that was also used for the MS search). In that sense, the peptide products were randomized. This simulated background database could then be compared to the database of experimentally identified peptide products. Thereby, we could verify whether the identification of spliced and non-spliced peptide characteristics (*e.g*., splice-reactant, intervening sequence and peptide lengths, as well as amino acid frequencies) arose from theoretical database structure – and thus were potential analysis artefacts - or from biochemical drivers of the catalytic reaction. In this study we made use of the simulated background database to investigate amino acid preferences of forward and reverse *cis*-spliced peptides.

### Mapping of peptide sequences. Identification of peptides containing N- or C-termini of substrates. Identification of spliced peptides with one amino acid long splice-reactant

Peptide sequences were mapped to a substrate sequence by exact string matching of the complete peptide product sequence. If this was not possible, the peptide product sequence was split into two splice-reactants at each possible position. Each pair of splice-reactants was then matched against the substrate sequence. If both splice-reactants could be matched to the substrate sequence, the respective locations within the substrate were recorded.

If a peptide sequence could be explained by multiple locations, all locations have been reported in the final database. However, when we computed frequency and features of product types, we applied the following rules: (i) if a sequence could be both a non-spliced and a spliced peptide, we defined it as non-spliced peptide; (ii) if a sequence could be both a *cis*-spliced and a *trans-*spliced peptide, we defined it as *cis*-spliced peptide; (iii) if a sequence could be both a forward *cis*-spliced and a reverse *cis*-spliced peptide, we defined it as forward/reverse *cis*-spliced peptide (*i.e*., multi-mapper *cis*-spliced peptide). Implications of such multi-mapper peptides, *i.e*., peptides that map to multiple locations in the substrate, are discussed below.

A peptide with several potential substrate origins was assigned to the category “peptides containing N- or C-termini of their cognate synthetic polypeptide substrate” only in case all possible peptide locations contained the substrate’s N- or C-terminus. Analogously, a peptide with several potential substrate origins was assigned to the category “spliced peptides with one amino acid long splice-reactant” only if none of the possible origins resulted in longer splice-reactants.

### Calculation of all possible *cis-*spliced and non-spliced peptide products to investigate length and presence of substrate’s N- or C-termini

The number of possible unmodified spliced and non-spliced peptides that could be derived from a protein sequence in sequence-agnostic fashion formed the theoretical sequence search space. The number *X* of non-spliced peptides of length *N* that could theoretically arise from a substrate of length *L* was:

$${X}_{non-spliced}=L-N+1$$

To derive the theoretical number of all spliced peptides, we defined four indices *i,j,k* and *n* that denoted the positions of the first (*i,j*) and second (*k,n*) splice-reactant, respectively. The corresponding number of peptides was calculated via summing over interval ranges that form valid spliced peptides. *Cis*-spliced peptides could be formed via forward or reverse ligation. The number of all forward *cis*-spliced peptides of length *N* that could theoretically arise from a substrate of length *L* was:

$${X}_{fwd.cis-spliced}=\mathop{\sum }\limits_{i=1}^{L-N}\mathop{\sum }\limits_{j=i+{L}_{ext}-1}^{N-{L}_{ext}+i-1}\mathop{\sum }\limits_{k=j+2}^{L-N+j-i+2}1=\frac{1}{2}\left(N-2{L}_{ext}+1\right)\left(L-N\right)\left(L-N+1\right)$$

*L*_*ext*_ denoted the minimal splice-reactant length and was set to 1 per default. In case a peptide was located at either of the substrate’s termini (*i* = 1 or *n* = *L*), the number of forward *cis*-spliced peptides was calculated according to:

$${X}_{fwd.cis-splicedattermini}=\left(N-2{L}_{ext}+1\right)\left(L-N\right)$$

Analogously, the number of theoretically possible reverse *cis*-spliced peptides was calculated as:

$${X}_{rev.cis-spliced}=\mathop{\sum }\limits_{k=1}^{L-N+1}\mathop{\sum }\limits_{n=k+{L}_{ext}-1}^{N-{L}_{ext}+k-1}\mathop{\sum }\limits_{i=j+1}^{L-N+n-k+2}1=\frac{1}{2}\left(N-2{L}_{ext}+1\right)\left(L-N+1\right)\left(L-N+2\right)$$

$${X}_{rev.cis-splicedattermini}=\left(N-2{L}_{ext}+1\right)\left(L-N+1\right)$$

To calculate the number of theoretical *trans*-spliced peptides in an *in vitro* scenario where a single synthetic polypeptide substrate was digested with purified proteasome, the following formula was derived:

$${X}_{trans-spliced}=-1+\frac{2}{3}{L}_{ext}^{3}+{L}_{ext}^{2}\left(-1-N\right)+\frac{5}{6}N+{N}^{2}-\frac{5}{6}{N}^{3}+L\left(-1+{L}_{ext}\left(2-2N\right)+{N}^{2}\right)+{L}_{ext}\left(\frac{7}{3}-3N+2{N}^{2}\right)$$

$${X}_{trans-splicedattermini}=N\left(N-2{L}_{ext}+1\right)$$

To note, the number of non-spliced peptides of length *N* that could be derived from either of the substrate’s termini was 2.

### InvitroSPI and invitroPB pipelines

**Fig. 2:**
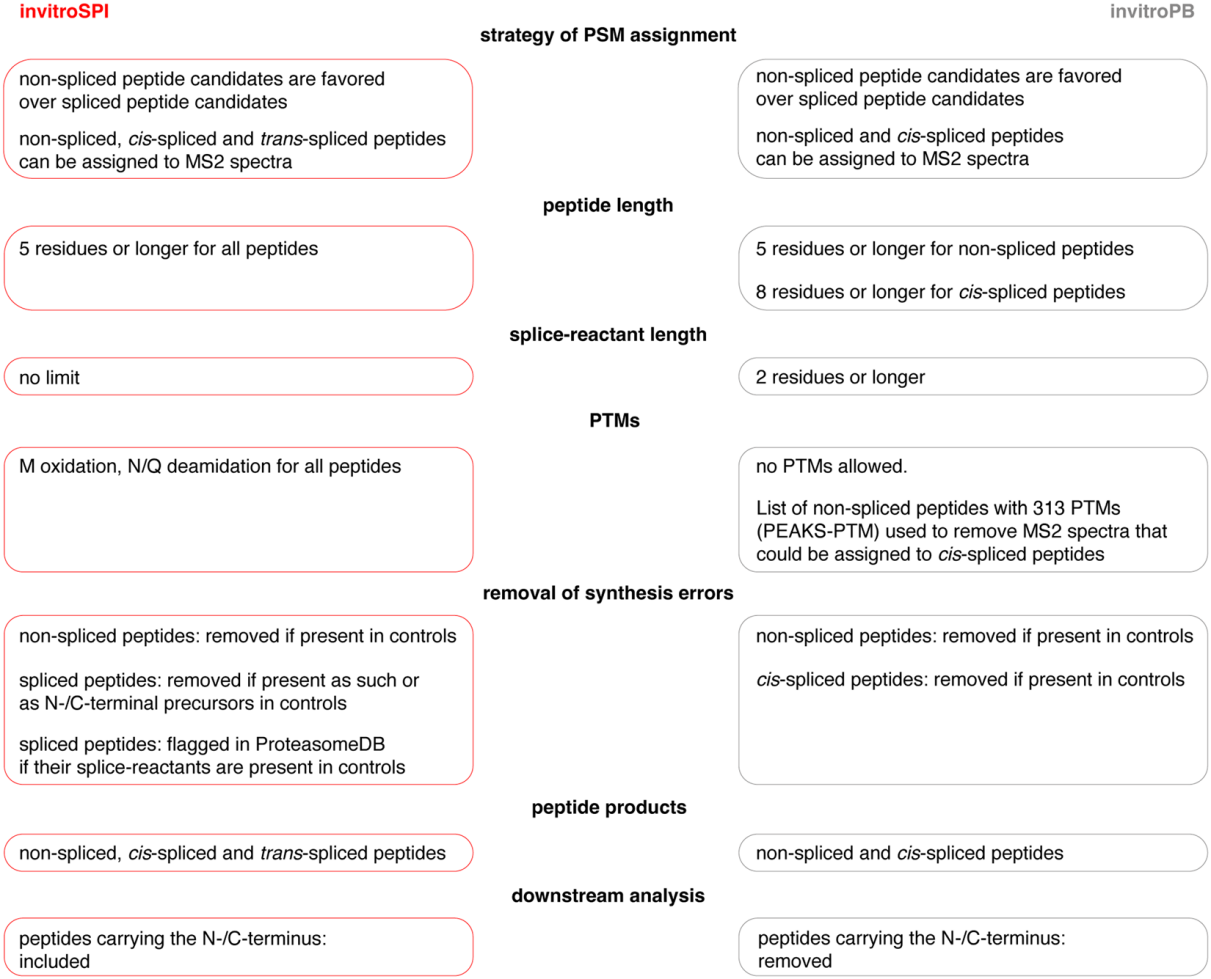
Difference in the peptide identification strategy and downstream analysis adopted by invitroSPI and invitroPB.

The computational pipelines of invitroSPI and invitroPB differ as follow (Fig. 2):

- Both invitroSPI and invitroPB adopted conservative approaches by favoring the assignment of non-spliced over spliced peptides to counteract the imbalance of the theoretical sequence search space (Fig. S1, S2). Indeed, the theoretical search space computed from the 80 substrate sequences of the whole dataset is 400-fold larger for spliced compared to non-spliced peptides, and significantly larger for *trans*- vs. c*is*-spliced peptides (Fig. S3). InvitroPB can identify only non-spliced and *cis*-spliced peptides, whereas invitroSPI can also identify *trans*-spliced peptides. Their inclusion in the final peptide product database could enrich the information that may be used to understand proteasome catalytic activities. InvitroSPI can identify peptide-spectrum matches (PSMs) that may be *trans*-spliced peptides, assign them if there is no better non-spliced candidate and the scan fulfills all quality criteria described above. Although this strategy may lead to a higher FDR for *trans*-spliced peptides compared to non-spliced peptides (see below), it may avoid the misassignment of MS2 spectra to non-spliced peptides, which, in reality, are *trans*-spliced peptides;
- InvitroSPI applies a general threshold of at least 5 amino acid length for all peptides, and therefore does not apply different restrictions of peptide length between product types. InvitroPB, on the contrary, sets a different minimal length threshold for *cis*-spliced (8 amino acids) and non-spliced (5 amino acids) peptide candidates;
- InvitroSPI allows the identification of spliced peptides with a splice-reactant length of one amino acid. These peptides could not be identified through invitroPB. To note, proteasomes can perform a second cleavage on a spliced peptide, thereby reducing the length of a splice-reactant to one amino acid after the PCPS reaction. This event was described *in vitro* and *in cellula* for a gp100-derived *cis*-spliced epitope by Michaux and colleagues^51^. That *cis*-spliced epitope was also demonstrated to be recognized by CD8^+^ T cells of melanoma patients^20^, thereby confirming that *cis*-spliced peptides with a one amino acid long splice-reactant can be produced by proteasomes, and in an amount sufficient to be presented and to trigger a CD8^+^ T cell response;
- InvitroSPI allows the identification of non-spliced and spliced peptides with two post-translational modifications (PTMs) – *i.e*., N/Q deamidation and M oxidation. On the contrary, the implemented invitroPB method does not allow the identification of peptides with PTMs, although it removes any query matched through PEAKS-PTM for the downstream *cis*-spliced peptide identification. PEAKS-PTM performs an open search of 313 PTMs, which could have similar statistical challenges as the identification of spliced peptides, since they both strongly increase the peptide sequence search space for MS2 spectrum assignment. In addition, many PTMs could not occur during the synthesis and *in vitro* digestions of the polypeptide substrates, such as ubiquitination or phosphorylation; therefore, we think that their prioritization over spliced peptides is not supported by biological evidence and may reduce the method’s recall of *cis*-spliced peptides (see below).
- Both invitroSPI and invitroPB adopt approaches to tackle the issue of the synthesis errors inherent in the synthetic polypeptides. The synthesis errors may appear as the product of PCPS if amino acids are skipped or added more than once during synthesis or may arise through hydrolysis of a contamination (here referred to as synthesis errors; Fig. 1f). In contrast to invitroPB method, invitroSPI adopts a more conservative approach since it removes spliced peptides not only if they are identified as such in control samples, but also if any longer spliced peptide containing the same splice-site is identified in control samples.

### Technical aspects of invitroSPI (Fig. S1)

The invitroSPI method is an improvement on the method previously described in Specht *et al*.^8^, which was developed to tackle the issue of synthesis errors and the large number of theoretically possible spliced peptides that could be derived from one substrate in the database. Briefly, MS RAW files were converted to MGF with ProteoWizard msconvert, using the vendor peak picking option. Data have been searched against a custom reference database containing all theoretically possible *cis*-spliced, *trans*-spliced and non-spliced peptides derived from the substrate of interest and with a minimal length of at least 5 amino acids. The custom reference databases were generated in FASTA format as previously described^63^. Briefly, we generated all possible spliced and non-spliced peptides as follows: (i) in the case of non-spliced peptides, by applying a single sliding window over the substrate sequence. The sliding window could vary in its size, reflecting a variable length of the peptide product; (ii) in the case of spliced peptides, we applied two sliding windows, which were *in silico* ligated if they could form a valid spliced peptide, as determined by their substrate origins.

The following variable modifications have been set whilst applying invitroSPI method to both non-spliced and spliced peptides: asparagine (N) and glutamine (Q) deamidation and methionine (M) oxidation. All ranked PSMs suggested by the Mascot Server for a single MS2 scan (query) were mapped to all potential origins in the substrate sequence, thereby considering the redundancy of leucine (L) and isoleucine (I) (see ‘Mapping of peptide sequences’ section above). Subsequently, PSMs have been evaluated based on product type (spliced vs non-spliced) and differences in ion scores to determine the most probable peptide sequence and origin. Scans that did not allow for the high-confidence identification of a single peptide were not assigned and removed from further analysis. For all PSMs, the mandatory condition for the peptide identification was: (i) the Mascot ion score was higher than 20, (ii) the Mascot q-value was lower than 0.05. In the case that the top-ranked peptide was a spliced peptide, it was considered a correct PSM if the difference in Mascot ion score between the first-ranked and the second-ranked peptide (either non-spliced or spliced) was larger than 30%, *i.e*., the delta score was larger than 0.3. This optimal delta score was determined by FDR estimation (see below, Fig. S4). In case there were several non-redundant sequences with identical scores identified, the scan was assigned only if there was a single, non-ambiguous non-spliced peptide among them that passed all other criteria mentioned above. This approach favors the assignment of non-spliced peptides over spliced peptides to counteract the imbalance of the large theoretical number of spliced and non-spliced peptides in the MS search space.

To select the best delta score for invitroSPI in the datasets investigated in this study, we applied invitroSPI to the PB dataset repeatedly, while varying the delta-score in a range from 0 to 0.5. The identified PSMs were subsequently compared to the predicted MS2 spectrum by application of Prosit^61^ and computation of spectral angles between normalized MS2 spectra for each product type. The spectral angle distribution for the identified non-spliced peptides represented the ‘gold-standard’ to which all other spectral angle distributions resulting from spliced peptides were compared to. If we assumed 1% False Discovery Rate (FDR) among the non-spliced peptides, we could determine a spectral angle cut-off, for which 1% of the non-spliced peptide PSMs fall below this cut-off and 99% of the non-spliced peptide PSMs fall above this cut-off. The same cut-off applied to spliced peptides allowed us to estimate the FDR for *cis*-spliced peptides, *trans*-spliced peptides or all spliced peptides compared to non-spliced peptides. For each investigated delta score, the FDRs for each product type were estimated and the delta score resulting in lowest FDR for spliced peptides was selected (*i.e*., delta score = 0.3; Fig. S4).

Spliced peptides generated by ligation of three or more fragments were not allowed and therefore are not included in our database.

As in Specht *et al*.^8^, for each substrate digestion, peptide synthesis artefacts identified in control samples (either 0 h digestion time or samples with substrates and no proteasomes) were removed as follows: any non-spliced peptide identified in control samples was removed from the final list of identified non-spliced peptides. Any spliced peptide in the control samples, containing the same splice-site as an identified peptide (thus, either identified as such or identified as a longer precursor in control samples) was removed from the final list of identified spliced peptides (Fig. 1f, Fig. S1).

InvitroSPI is available as a user-friendly and readily executable tool on GitHub (see Code Availability section).

### Technical aspects of invitroPB (Fig. S2)

We implemented Paes’ method based on the information provided in the original publications and source code^14,57^. Briefly, in invitroPB, MS data were first searched against a reference custom database containing only a given substrate sequence using PEAKS DB (PEAKS v10.5). Additionally, an open search for PTMs using PEAKS PTM (313 variable PTMs included) was performed. Although PSMs of non-spliced peptides with PTMs were not further considered, their corresponding MS2 spectra were dismissed and not further investigated. To note, while the original method described by Paes *et al*.^57^ discarded all PTM-labelled non-spliced peptides during assignment, our invitroPB implementation recorded PTM-labelled non-spliced peptides. Those peptides were, however, not considered for downstream analyses; recording them served solely the purpose of dissecting the outcomes of the steps of method’s strategy.

MS2 spectra not assigned as non-spliced peptides (with or without PTMs) with 5% PEAKS-computed FDR were re-searched using PEAKS De novo (without PTMs), which also converted all possible I to L amino acids. For the following analysis the top 100 *de novo* candidate sequences per MS2 spectrum with an ALC score equal or larger than 50 were exported, but only those *de novo* sequences within the top 5 ALC scores were further considered. All *de novo* sequences within the top 5 ALC scores were screened to determine if they could be generated through PCPS from the given substrate sequence upon exchange of all Is with Ls. All sequences that could be explained as non-spliced peptide sequences were removed. Among the remaining sequences, the implemented method computed those that could be *cis*-spliced peptides with splice-reactant length larger than 1 amino acid and a peptide length larger than 7 amino acids, which were then kept. Therefore, invitroPB could not identify *trans*-spliced peptides, *cis*-spliced peptides with a 1 amino acid long splice-reactant, and *cis*-spliced peptides with a length smaller than 8 amino acids. If more than one *cis*-spliced peptide candidate per MS2 spectrum was listed, only the peptide sequence with highest ALC score was kept and considered as the assigned sequence to that MS2 spectrum. Non-spliced and *cis*-spliced peptides, identified in the samples containing substrates but not proteasomes, were removed to exclude peptide products that may arise from peptide synthesis errors (Fig. 1f). As downstream filtering steps, Paes *et al*.^57^ and invitroPB did not further consider non-spliced and *cis*-spliced peptides carrying the N- or C-termini of synthetic polypeptide substrates within their sequence.

As technical validation of our implementation, we applied invitroPB to the PB dataset, and obtained a partially different non-spliced and *cis*-spliced peptide list than published by Paes *et al*.^57^ (Fig. S5a). This difference could in part be explained by a different PEAKS version applied by Paes *et al*.^57^ – *i.e*., PEAKS v8.0 – and by invitroPB (PEAKS v10.5)^64^. Indeed, when we applied invitroPB method - using either PEAKS v8.5 or v10.5 – on *in vitro* digestions of six substrates of the PB dataset, we observed some differences in the non-spliced and *cis*-spliced peptides list (Fig. S5b). Similarly, we noted a difference between the spliced and non-spliced peptides published by Paes *et al*.^57^ and the spliced and non-spliced peptides derived using invitroPB method using PEAKS v8.5 (Fig. S5c), which could have been explained in a *corrigendum* by the same authors published during the revision of the current manuscript^65^. Nonetheless, in invitroPB, which used the better performing PEAKS v10.5^66^, the pipeline and filtering steps of the original study were conserved, which allowed a proof-of-principle comparison of invitroPB and invitroSPI.

## Data Records

The MS files (.RAW, .mgf and search result files) of the Specht dataset^8^ are available at the PRIDE repository^67^ with the dataset identifier PXD016782^68^.

The MS .RAW and .mgf files of the PB dataset are available at the PRIDE repository with the dataset identifier PXD021339 and PDX025893^57^.

The MS files (.RAW, .mgf and search result files) of the gp100 Fusion dataset are available at the PRIDE repository with the dataset identifier PXD025995^69^. The reference custom databases that contain all theoretically possible spliced and non-spliced peptides and that were used to perform the MS search are available in a Figshare repository^70^.

The final database – *i.e*. ProteasomeDB - with all identified spliced and non-spliced peptide products, as well as their substrate sequences, is provided as CSV file, and is available in a Figshare repository^70^. In ProteasomeDB, all Is of identified peptide products were replaced by Ls, whereas the substrate sequence contains the original I/L amino acids.

All ‘online-figures’ and ‘online-tables’ reported are available in a Figshare repository^70^.

## Technical Validation

### Comparison and validation of invitroSPI and invitroPB methods in gp100 Fusion dataset

Our aim was to create ProteasomeDB – a database of non-spliced and spliced peptides produced *in vitro* by proteasomes and reliably identified by a single method with the highest recall of peptide products. Hence, we initially compared invitroSPI and invitroPB, to then select a single method and apply it to the whole dataset, thereby generating ProteasomeDB. Due to its dependence on *de novo* peptide sequencing, which relies on high-precision MS data, invitroPB could not be applied to the vast majority of digestions in the Specht dataset. Therefore, we initially validated and compared invitroSPI and invitroPB through the analysis of the PB dataset and the gp100 Fusion dataset by investigating methods’ features and performances. We put particular attention in dissecting the several filtering steps of the two methods (Fig. 2, Fig. S1-S2) and their impact on PSM identifications.

**Fig. 3:**
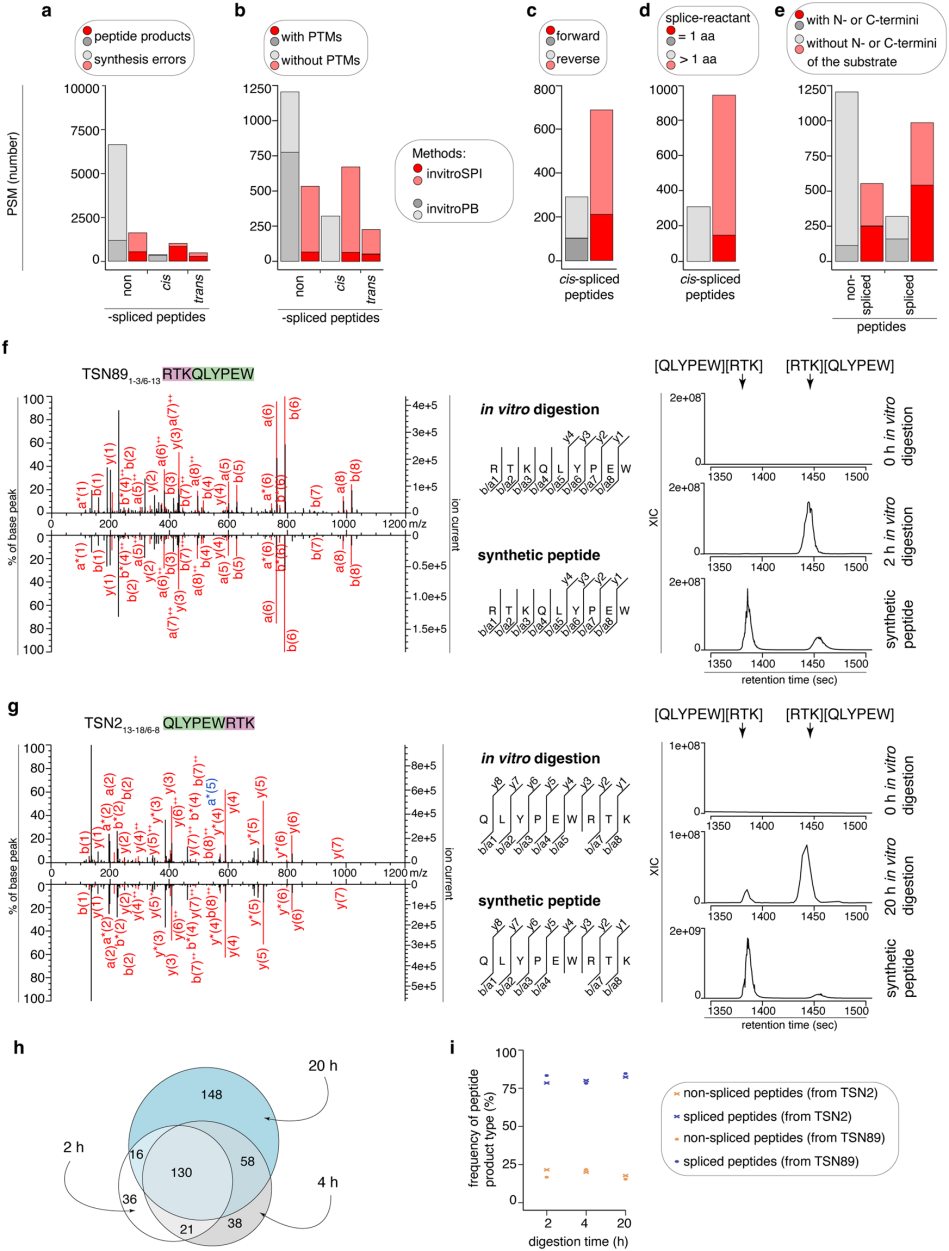
Comparison of invitroSPI and invitroPB methods applied to the gp100 Fusion dataset. **a**–**e**) Number of PSMs assigned to: (**a**) non-spliced, *cis*-spliced, *trans*-spliced peptides, and related synthesis error peptides, (**b**) PTM-labelled peptides, (**c**) forward and reverse *cis*-spliced peptides, (**d**) spliced peptides with one amino acid long splice-reactant, (**e**) spliced peptides containing substrate’s N- or C-termini. Assignment was carried out by applying invitroSPI and invitroPB methods to *in vitro* digestions of TSN2 and TSN89 substrates with proteasomes. PTM-modified non-spliced peptides identified by PEAKS-PTM are reported, although they are not kept in the final list of identified peptides by invitroPB. In invitroSPI identifications, PTM-modified peptides are included. In (**b-e**), PSMs assigned to synthesis errors have been removed. In (**c**), forward/reverse *cis*-spliced peptides, *i.e*. multi-mapping *cis*-spliced peptides, are not shown. **f,g**) MS2 spectra of the *cis*-spliced epitopes (**f**) [RTK][QLYPEW] and (**g**) [QLYPEW][RTK] identified in *in vitro* digestions of (**f**) TSN89 and (**g**) TSN2 substrates, and of their cognate synthetic peptides. Detected *m/z* and charges in the MS2 spectra shared between *in vitro* digestion samples and synthetic peptides are indicated in red. Other assigned *m/z* are indicated in blue. In MS2 spectra, charged b-, a- and y-ions are reported. Double charged ions are marked as ^++^. Ions’ neutral loss of ammonia is symbolized by *. Extracted ion chromatograms of target peptides in *in vitro* digestion and synthetic peptides are plotted in the right panels and indicate matching retention times and absence of a biologically meaningful peak in the 0 h digestion. MS ion chromatograms correspond to the m/z = 610.80–610.84 (+2; **f**) and 407.53–407.57 (+3; **g**). **h**) number of unique peptide sequences identified by invitroSPI in the gp100 Fusion dataset shown for 2 h, 4 h and 20 h. **i**) frequency of spliced and non-spliced peptides over time identified by invitroSPI in the gp100 Fusion dataset comprising two substrates. In (**a-e,h-i**) *in vitro* digestion samples (0, 2, 4, 20 h) and cognate synthetic peptides were measured by Orbitrap Fusion Lumos (KCL-CEMS) by using the same MS method. For MS2 spectrum references, (**f**): file 20210422_WB2_2h_TSN89_FusionCEMS, charge + 2, scan 5897 (upper panel); file 20210422_GP100_mix_FusionCEMS, charge + 2, scan 5208 (lower panel). (**g**): file 20210422_WA4_20h_TSN2_FusionCEMS, charge + 3, scan 6115 (upper panel); file 20210422_GP100_mix_FusionCEMS, charge + 3, scan 4936 (lower panel).

The gp100 Fusion dataset contained two substrates, TSN2 and TSN89 (Fig. 1e). TSN89 is a subsequence of TSN2, which is the gp100_35-53_ sequence. Two spliced epitopes immunogenic in melanoma patients have been identified within this sequence^2,20^, including the first *cis*-spliced epitope initially described by Vigneron and colleagues^2^. We measured the *in vitro* digestions (0, 2, 4, 20 h) of the synthetic polypeptide substrates with human 20 S standard proteasomes through highly-sensitive Orbitrap Fusion Lumos mass spectrometers. We then applied invitroSPI and invitroPB method to the MS files. For each scan, both methods aim to assign the most likely PSM. A single unique peptide sequence can be assigned to multiple MS2 scans. InvitroSPI identified a larger number of unique non-spliced peptides, *cis*-spliced and *trans*-spliced peptides as compared to invitroPB (Table 2). This generally reflected what we observed at PSM level, albeit invitroPB method assigned more PSMs to non-spliced peptides compared to invitroSPI (Fig. 3a). InvitroSPI discarded, as synthesis errors, hundreds of PSMs of potential *cis*-spliced peptides, whereas invitroPB method eliminated only 10 of them (Fig. 3a, Online-Table 2). Upon removal of the synthesis errors, over 700 PSMs were discarded by invitroPB method because they were suggested to be PTM-modified non-spliced peptides by PEAKS-PTM (Fig. 3b). One of them was assigned to a spliced peptide sequence by invitroSPI. Both methods assigned fewer PSMs to forward than reverse *cis*-spliced peptides (Fig. 3c). InvitroSPI assigned over a hundred PSMs to spliced peptides with a one amino acid long splice-reactant, and over 500 PSMs to spliced peptides containing N- or C-termini of the substrates. These peptides were not identified by invitroPB analysis because of the different strategy of this method (Fig. 3d,e and Online-Table 2).

**Table 2:** Number of unique peptides identified in the various datasets by applying different identification methods.

	Datasets analyzed by invitroSPI				Datasets analyzed by invitroPB	
	gp100 Fusion	PB	Specht	whole	gp100 Fusion	PB
**Peptide types:**						
Non-spliced	68	1,196	4,288	5,493	46	1,185
*Cis*-spliced	275	1,403	4,915	6,453	185	1,060
*Trans*-spliced	115	814	3,781	4,685	0	0
Forward *cis*-spliced	96	838	2,828	3,716	71	701
Reverse *cis*-spliced	174	476	1,876	2,435	101	316
Forward/reverse *cis*-spliced (multi-mapper)	5	89	211	302	13	43
Spliced with 1 amino acid splice-reactant	67	409	943	1,390	0	0
Non-spliced with N- or C-terminal residues	11	298	634	932	1	104
spliced with N- or C-terminal residues	204	1,530	5,247	6,876	89	653

Number of unique peptides identified through the application of invitroSPI and invitroPB to the PB, Specht and whole datasets. In this table, all substrates, all proteasome types, and time points, have been included.

We also compared the MS2 spectra assigned by the two identification methods to the MS2 spectra of a pool of synthetic non-spliced, *cis*-spliced, and *trans*-spliced peptides, which have been previously investigated^2,5,9,20,29^ (Table S1). Among these peptides, both invitroSPI and invitroPB method identified many non-spliced and *cis*-spliced peptides, in addition to *trans*-spliced peptides, which could be identified only by invitroSPI (Online-Fig. 1, Online-Table 2). Both methods identified the *cis*-spliced epitopes TSN89_1-3/6-13_ (gp100_40-42/47-52_) [RTK][QLYPEW] and TSN2_13-18/6-8_ (gp100_47-52/40-42_) [QLYPEW][RTK] (Fig. 3f,g), which have been proven to be produced by proteasomes and presented by HLA-I complexes of cancer cell lines^2,5,9,20,29^.

When considering the single time points, *i.e*., 2, 4 and 20 h, of the digestion kinetics, invitroSPI identified 458 unique peptides upon removal of the synthesis errors whereas invitroPB identified 231 unique peptides (Table 2). Although overall more peptides were identified at later time points compared to earlier time points by invitroSPI (Fig. 3h), the frequency of spliced and non-spliced peptides remained constant over time (Fig. 3i), in agreement with our previous observation in Specht *et al*.^8^.

### Comparison and validation of invitroSPI and invitroPB methods in PB dataset

**Fig. 4:**
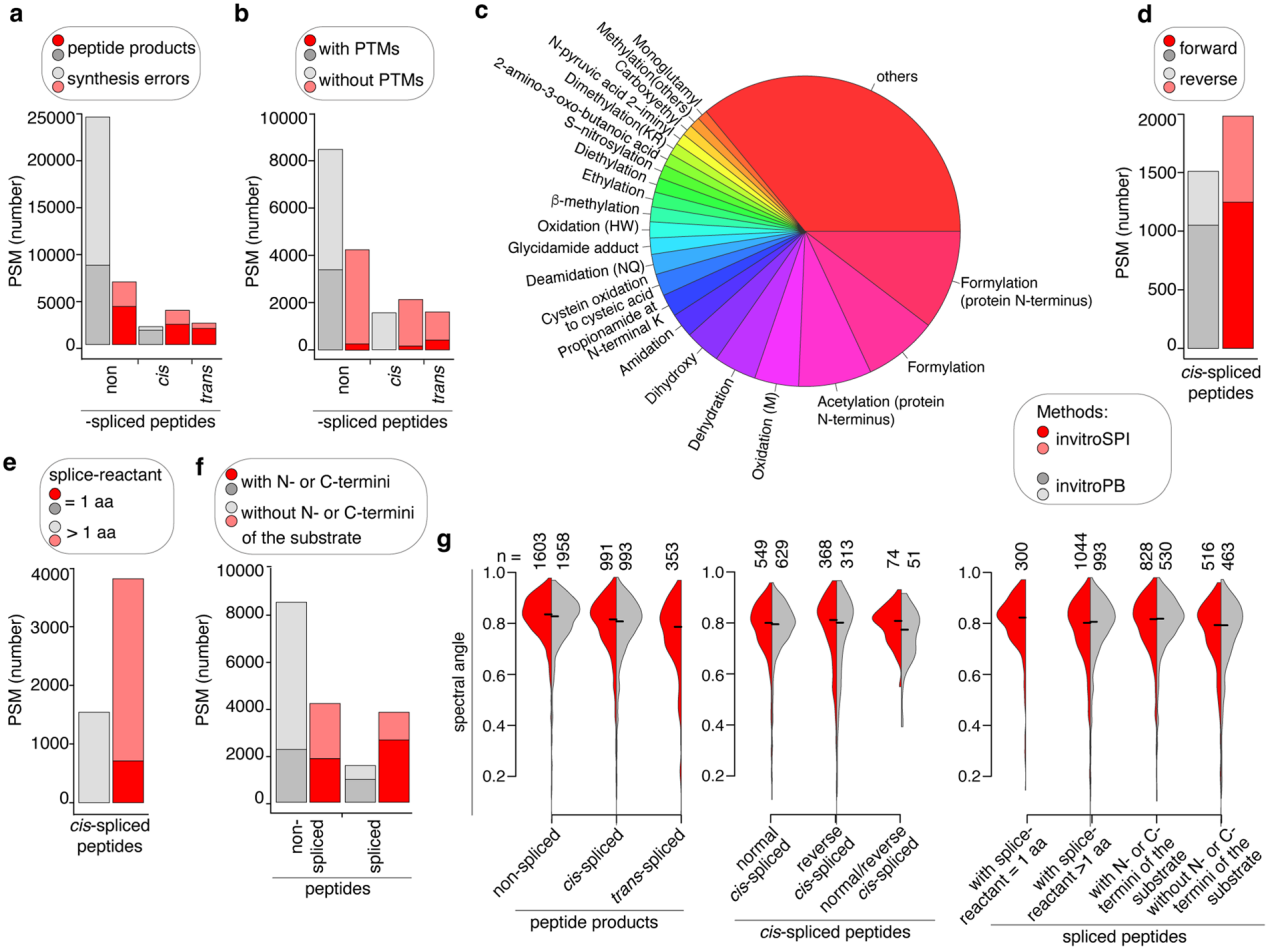
Comparison of invitroSPI and invitroPB methods applied to the PB dataset. (**a,b**) Number of PSMs assigned to: (**a**) non-spliced, *cis*-spliced, *trans*-spliced peptides, and either related synthesis error peptides, or (**b**) PTM-labelled peptides. (**c**) Frequency of PTMs among PTM-labelled non-spliced peptides suggested by PEAKS-PTM as part of invitroPB. (**d-f**) Number of PSMs assigned to: (**d**) forward and reverse *cis*-spliced peptides (multi-mapper forward/reverse *cis*-spliced peptides are not shown), (**e**) spliced peptides with one amino acid long splice-reactant, and (**f**) spliced peptides containing substrate’s N- or C-termini. Assignment was carried out by applying invitroSPI and invitroPB methods to the PB dataset. In invitroSPI-identified peptides, PTM-modified peptides are also included. In (**b**) and (**d-f**), PSMs assigned to synthesis errors have been removed. (**g**) Spectral angle distribution computed between measured and predicted MS2 spectra identified by invitroSPI (red) and invitroPB methods (grey). Only PSMs of unmodified non-spliced and spliced peptide that do not contain any cysteine (C) residues, do not exceed a charge of 6 and are 7–12 amino acid long are here included, since Prosit cannot predict PTM-modified peptide’s MS2 spectra and Prosit performance is influenced by peptide length (Fig. S6). In the violin plots, horizontal black lines represent the median. The number of PSMs for each group is reported. In (**a-g**), *in vitro* digestion samples (2 h and 20 h digestions with proteasomes and 20 h without proteasomes) were measured by Orbitrap Fusion Lumos (Oxford proteomics centre).

To compare and evaluate the performance of the two methods on a larger dataset, we next applied invitroSPI and invitroPB to the PB dataset of 25 synthetic polypeptides, digested for 2 h and 20 h with 20 S standard proteasomes (Fig. 1e). Control samples were left for 20 h without proteasomes, but otherwise in the same conditions of the digestion kinetics. Overall, the analysis of the PB dataset confirmed what was observed on the smaller gp100 Fusion dataset. Indeed, invitroSPI identified more unique non-spliced, *cis*-spliced and *trans*-spliced peptides than invitroPB (3,413 peptides identified by invitroSPI and 2,245 peptides identified by invitroPB; Table 2), which was also observed at PSM level (Online-Table 3). As observed in the analysis of the gp100 Fusion dataset, both methods discarded PSMs of potential *cis*-spliced peptides as synthesis errors, although this filtering step was more stringent in invitroSPI (Fig. 4a). After synthesis error removal in both methods, invitroPB, using PEAKS-PTM, identified and discarded over 3,000 putative PTM-labelled non-spliced peptides (Fig. 4b). InvitroSPI assigned around 250 PSMs of those discarded PSMs to spliced peptides. A distribution of PTMs identified at the PEAKS-PTM step of invitroPB is shown in Fig. 4c. Both methods assigned more PSMs to forward than reverse *cis*-spliced peptides in the PB dataset (Fig. 4d). In contrast to invitroPB, over 700 PSMs were assigned by invitroSPI to spliced peptides with one amino acid long splice-reactant, and over 2,000 PSMs to spliced peptides containing N- or C-termini of the substrates (Fig. 4e,f).

**Fig. 5:**
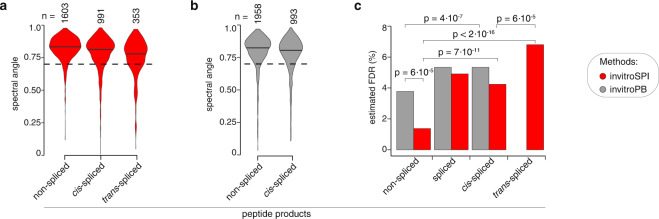
FDR estimation for invitroSPI and invitroPB in PB dataset. (**a,b**) Spectral angle distribution of non-spliced, *cis*-spliced and *trans*-spliced peptide identified by either (**a**) invitroSPI or (**b**) invitroPB in the PB dataset. (**c**) Estimated FDRs based on spectral angle distributions, choosing a spectral angle cut-off of 0.7 (dash line) reported in (**a,b**).The bars represent the relative frequency of PSMs below the cut-off in each peptide strata. Statistically significant p values < 0.05 (two-samples Wilcoxon test) are reported in (**c**), and they refer to the comparison of the spectral angle distribution shown in (**a,b**).

The two methods showed a high similarity between measured and predicted MS2 spectra (reflected by high spectral angles) for all peptide groups (Fig. 4g, Fig. 5, Fig. S6, Online-Figs. 2–3), thereby confirming their reliable and comparable identification of PSMs. MS2 spectra were predicted by applying Prosit^61^. In this analysis, we considered non-spliced and spliced peptides which did not contain any cysteine residues (C), did not exceed a charge of +6 and were between 7 and 12 amino acids long, because Prosit showed a progressive decrease of its prediction performance on non-spliced peptides for longer peptides and/or peptides with higher charges (Fig. S6), in agreement with previously described analyses^61^.

As last step of method validation, we estimated the FDRs of invitroSPI and invitroPB in PB dataset by using the spectral angle analysis. We chose a spectral angle cut-off of 0.7 as approximative threshold to estimate the FDRs, with high-quality PSMs having a spectral angle above this threshold (Fig. 5a). The percentage of PSMs below this cut-off and identified as non-spliced peptides by invitroSPI was 1.4%, which could be interpreted as an estimated 1.4% FDR (Fig. 5b). By applying the same strategy for the computation of the FDR of spliced peptides, we estimated that invitroSPI had a 4.2% FDR for *cis*-spliced peptides and a significantly larger 6.8% FDR for *trans*-spliced peptides (Fig. 5b). For invitroPB, the estimated FDRs were higher than those of invitroSPI for non-spliced peptides (statistical significance was reached only for non-spliced peptides). Indeed, 3.8% of the PSMs assigned to non-spliced had a spectral angle below 0.7, which increased to a 5.3% for *cis*-spliced peptides (Fig. 5c). The estimated FDRs for both non-spliced and spliced peptides identified by both methods should be considered critical in the use and evaluation of ProteasomeDB.

### ProteasomeDB – a non-spliced and spliced peptide product database computed through the application of invitroSPI on the whole dataset

Our comparison of invitroSPI and invitroPB on these two datasets showed that both methods successfully identified non-spliced and *cis*-spliced peptides produced by 20 S proteasomes in *in vitro* digestions of synthetic polypeptides. However, invitroSPI systematically identified more unique non-spliced and *cis*-spliced peptides per substrate than invitroPB (Table 2), in addition to the identification of *trans*-spliced peptides. The FDR estimation hinted toward a lower FDR for invitroSPI compared to invitroPB for both non-spliced and *cis*-spliced peptides (Fig. 5). Furthermore, invitroSPI was - contrary to invitroPB – applicable to various kinds of MS and does not rely on high-precision instruments. Therefore, invitroSPI represented a suitable method for the analysis of the whole dataset of proteasome-catalyzed *in vitro* digestions of synthetic polypeptides.

Through the application of invitroSPI on the whole dataset of 80 substrates - derived from the combination of the PB dataset (25 substrates), the Specht dataset (55 substrates), and the gp100 Fusion dataset (TSN2 and TSN89 substrates) (Fig. 1e) - we identified non-spliced (n = 5,493), *cis*-spliced (n = 6,453) and *trans*-spliced (n = 4,685) unique peptides (Table 2). They represented 33% (non-spliced peptides), 39% (*cis*-spliced peptides), and 28% (*trans*-spliced peptides) of the 16,631 unique peptides of the whole peptide product database (Table 2).

**Fig. 6:**
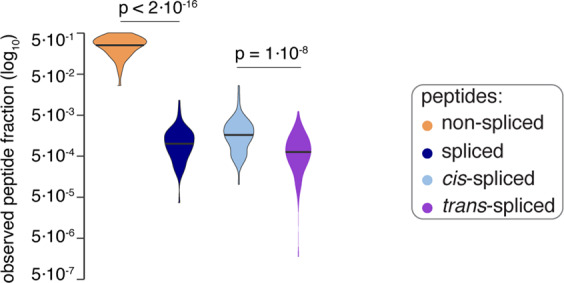
Generation efficiency of spliced and non-spliced peptides. Violin plots show the distribution of generation efficiencies for peptide hydrolysis and splicing. Generation efficiencies were calculated as the number of detected over the number of theoretically possible peptides for each substrate. Calculations were carried out on the peptide products and substrate sequences in the whole dataset digested with 20 S standard proteasome (80 substrates). The generation efficiency differs significantly between spliced and non-spliced peptides and, among spliced peptides, between *cis*- and *trans*-spliced peptides. Significant p values of a two-samples Wilcoxon test are reported.

While the overall frequency of spliced peptides may appear high at first glance, it is worthwhile considering the number of theoretical peptide sequences here. The generation efficiency on qualitative level takes the theoretical search space - *i.e*. the number of peptides that could be theoretically produced by proteasomes – into account (Fig. S3). If we defined the generation efficiency as number of detected peptides over the theoretical number of peptides in each peptide product type, PCPS had, on average, a 280-fold lower generation efficiency than peptide hydrolysis in the whole dataset. Indeed, on average per substrate, 27.2% of all non-spliced peptides were produced by 20 S proteasomes and detected by MS. In contrast, 0.16% of all theoretically *cis*-spliced and 0.06% of all theoretically *trans*-spliced peptides were produced by 20 S proteasomes and detected by invitroSPI (Fig. 6).

Among the unique peptides per substrate reported in the new ProteasomeDB, 1,031 non-spliced, 2,549 *cis*-spliced and 1,517 *trans*-spliced peptides were not reported in Specht and Paes databases of peptide products. In addition, 4,462 non-spliced, 3,904 *cis*-spliced and 3,168 *trans*-spliced peptides originally reported in Specht and Paes databases of peptide products were confirmed by the application of invitroSPI to the cognate datasets, bearing in mind that invitroPB could not identify *trans*-spliced peptides, which therefore were not detected in the original Paes databases of peptide products (Fig. S7a–c). To note, in this study we reported the number of unique peptides per substrate. Therefore, since Specht and PB datasets had no common substrates, they also had no common unique peptides per substrate. We adopted this strategy because we speculated that further analysis and eventual prediction of proteasome-catalyzed peptide hydrolysis and peptide splicing would, in most cases, consider the peptide sequence as well as its substrate origin. ProteasomeDB structure, however, also allows to obtain a list of unique peptide sequences regardless of their substrate origin, depending on the user’s choices and analysis goals.

### Illustrative analysis of the ProteasomeDB and the whole dataset: focus on 20 S standard proteasome and early time point digestions

So far, we selected and compared subsets of the whole dataset, as well as the outcome of different identification methods. ProteasomeDB (generated through the application of invitroSPI on the whole dataset) could, however, be large enough to carry out analyses on the catalytic nature of proteasome-catalysed peptide splicing and hydrolysis. As proof of principle, we here analyzed *in vitro* digestions carried out for 2/4 h with 20 S standard proteasomes and their corresponding controls. The analysis of these time points, for instance, could minimize the peptide product re-entry events in proteasomes; and the focus on 20 S standard proteasome digestion could be a strategy to limit the variance due to the different dynamics of proteasome isoforms^33^. In addition, in this illustrative analysis, we compared the features of the unique peptides either identified by applying invitroSPI and invitroPB methods to the 2 h PB dataset (24 substrates), or by applying invitroSPI to the 4 h Specht dataset (47 substrates) and the whole 2/4 h dataset of 71 substrates (white inlets in Fig. 1e). A comparison of invitroSPI with invitroPB on the whole 2/4 h dataset of 71 substrates could not be carried out, because invitroPB required high-precision MS data due to its dependence on de novo peptide sequencing, and many substrate digestions present in Specht dataset were measured by MS instruments with lower precision (Fig. 1e).

**Fig. 7:**
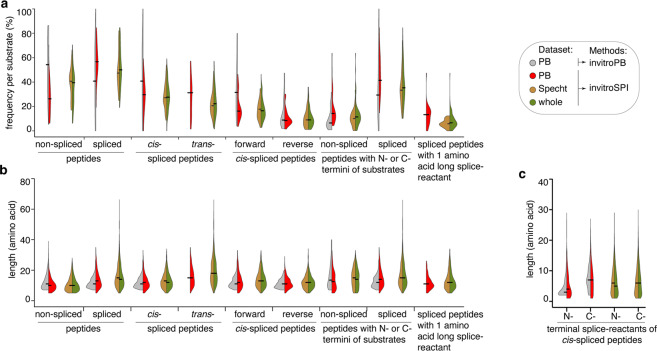
Features of unique peptides identified in all datasets. (**a,b**) Frequency (**a**) and length (**b**) of unique peptides per substrate. **c**) Length of N- and C-terminal splice-reactant of *cis*-spliced peptides that could unequivocally be assigned to a single position within a substrate. In (**a–c**), analysis has been carried out in the 2/4 h *in vitro* digestions with 20 S standard proteasomes, derived from the PB dataset (24 substrates) analyzed by invitroSPI and invitroPB, as well as from the Specht dataset (47 substrates) and the whole dataset (71 substrates) analyzed by invitroSPI. Here, PTM-tagged peptides identified by invitroSPI are added to the unmodified peptides. In (**a-c**), all peptides that could not be unambiguously annotated as either forward or reverse *cis*-spliced peptides (*i.e*. the multi-mapper forward/reverse *cis*-spliced peptides) were removed. Spliced peptides containing a single amino acid residue splice-reactant or the substrate’s N- or C-termini were labelled as such only if that was the only explanation out of all possible peptide origins within the polypeptide substrate. In (**c**), multi-mapper peptides that could be assigned unambiguously to a spliced peptide type were subsequently checked for the length of their splice-reactants. Among multi-mapper spliced peptides, only those that had a single and unambiguous splice-reactant length are included.

In this illustrative analysis, by applying invitroSPI to the PB dataset, we identified more unique *cis*-spliced (and of course *trans*-spliced) peptides than invitroPB, both considering the total number of unique peptides (Table 3) and the relative frequency of peptides per substrate (Fig. 7a). By applying invitroSPI to all three investigated datasets, we identified *cis*-spliced and *trans*-spliced peptides with a similar frequency (Table 3, Fig. 7a). InvitroSPI identified a sizeable portion of non-spliced and spliced peptides that contained the N- or C-termini of the substrates (Fig. 7a–c). These peptides were excluded in the analysis carried out by Paes *et al*.^57^, with consequences discussed below (Fig. 7a,b). InvitroSPI also identified a sizeable portion of spliced peptides with a one amino acid long splice-reactant, which could not be identified by invitroPB (Fig. 7a). Through the application of invitroSPI to the PB dataset, we did not observe a narrower length distribution of *cis*-spliced compared to non-spliced peptides (Fig. 7b), which was described by *Paes et al*.^57^. In all datasets analyzed by invitroSPI, non-spliced peptides were, on average, shorter than *cis*-spliced peptides, in contrast to what was described by Paes *et al*.^57^. Furthermore, in the whole dataset analyzed by invitroSPI, *cis*-spliced peptides were shorter than *trans*-spliced peptides (Fig. 7b), in agreement with what was previously described^8^. Because of multi-mapper spliced peptides and the features of the simulated background databases (see below), we avoided a more in-depth analysis of spliced peptide features. However, since Paes *et al*.^57^, suggested that the length of the N- and C-terminal splice-reactants of *cis*-spliced peptides differed, we preliminary investigated this aspect, focusing only on *cis*-spliced peptides that could be unequivocally assigned to a unique splice-reactant length. Although both methods identified *cis*-spliced peptides with, on average, shorter N-terminal spliced-reactants than the C-terminal ones in the PB dataset, this phenomenon was not confirmed in the larger Specht dataset and in the whole dataset. Indeed, in these two largest datasets analyzed through invitroSPI, N- and C-terminal splice-reactants of *cis*-spliced peptides had a similar length distribution (Fig. 7c). As discussed below, however, for an unbiased analysis, all biochemical characteristics of peptide product types should be compared to a simulated background database, to identify features that are specific for peptide hydrolysis and peptide splicing reactions.

**Table 3:** Number of unique peptides identified by applying different identification methods and focusing on 2/4 h digestions with 20 S standard proteasomes.

	Datasets analyzed by invitroSPI				Datasets analyzed by invitroPB	
	gp100 Fusion	PB	Specht	whole	gp100 Fusion	PB
**Peptide types:**						
**Non-spliced**	54	823	2,996	3,837	40	864
***Cis*-spliced**	171	759	2,363	3,240	101	617
***Trans*-spliced**	77	410	2,058	2,536	0	0
**Forward** ***cis*-spliced**	70	451	1,400	1903	43	404
**Reverse** ***cis*-spliced**	100	258	868	1,191	53	186
**Forward/reverse** ***cis*-spliced (multi-mapper)**	1	50	95	146	5	27
**Spliced with 1 amino acid splice-reactant**	37	232	456	711	0	0
**Non-spliced with N- or C-terminal residues**	10	224	514	740	1	88
**spliced with N- or C-terminal residues**	148	839	2,859	3,800	54	383

Number of unique peptides identified through the application of invitroSPI and invitroPB to the PB, Specht and the whole datasets. In this table only substrates digested with 20 S standard proteasomes for 2/4 h have been included.

### Potential pitfalls in data analysis: overview

For an appropriate investigation of sequence motifs and features of non-spliced and spliced peptides produced by proteasomes during the degradation of synthetic polypeptides, some factors play, in our opinion, a pivotal role: (i) the amino acid frequency should be normalized against an appropriate simulated background database to account for biases in the substrate amino acid composition; (ii) the database of identified peptides and digested substrates should be large enough to account for the large number of possible amino acid combinations; (iii-vi) non-spliced and spliced peptide identification algorithms could bias the features of the identified peptide pools, and, hence, methodological limitations should be considered during the analysis; (vii) for many spliced peptides, multiple splice-reactant locations are possible (multi-mapper peptides), thereby impinging upon the confidence in the computation of the features of splice-reactants, intervening sequences and PCPS splice-sites.

### Potential pitfalls in data analysis: (i) normalization strategy

One potential use case of ProteasomeDB is the analysis of amino acid preferences at the splice sites, *i.e*. sP1 and sP1’ (the two amino acid residues that are ligated together during PCPS; see Fig. 1b–d). In such analysis one should carefully consider the expected amino acid frequencies in sP1 and sP1’ observed by chance due to the limited sequence variety and amino acid composition of the substrates studied.

**Fig. 8:**
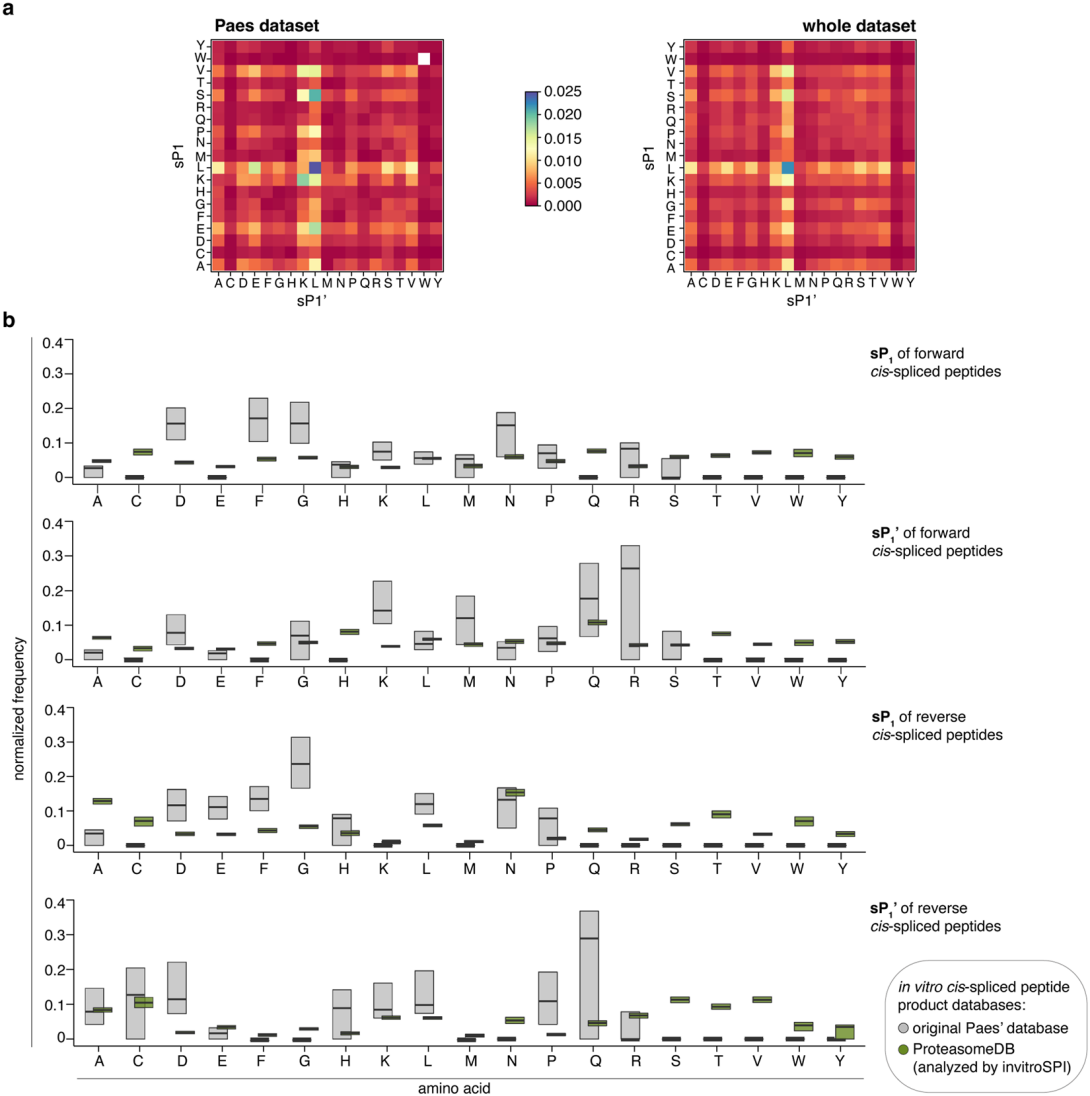
Potential pitfalls in data analysis related to peptide product database size. (**a**) Normalization strategies. Heatmaps display the joint frequency of amino acid combinations at the splice-site (formed by sP1 and sP1’) in the simulated background databases normalized by the amino acid frequency of the investigated substrates. Simulated background databases were computed from the PB dataset (n = 25 substrates) and from the whole dataset (n = 80 substrates). Frequencies were then normalized by the frequency of the amino acids within the substrate sequences. White spots indicate combinations that are impossible to derive from the given set of substrate sequences. Low frequencies are depicted in red, whereas high frequencies are shown in blue. (**b**) Amino acid frequencies at sP1 and sP1’ sites of forward and reverse *cis*-spliced peptides in the whole database of unique peptide products identified through invitroSPI, as well as those sequences originally published by Paes *et al*. The frequency in the true dataset was normalized by the frequency of the respective simulated background database as well as by the sum of all values. To verify the robustness of the frequency estimation, 200 bootstrap iterations were performed, each time sampling 80% of the splice-sites. The 90% confidence intervals of the resulting frequency estimations are displayed. Large confidence intervals indicate low robustness of the frequency estimation.

To this end, we computed the joint amino acid frequencies at sP1 and sP1’ based on the theoretical possible spliced peptides that could be derived from all studied substrates (simulated background database). The resulting frequency matrix represented the splice site background distribution (Fig. 8a), which in part reflected the natural amino acid frequency in the studied substrates. This non-uniform background distribution must be considered when analyzing *in vitro* digested spliced and non-spliced peptide products generated from polypeptides, especially when dealing with a small peptide product database with limited sequence diversity. Therefore, we suggest that all observed amino acid frequencies have to be normalized by their respective frequency in a simulated background database, and not only by amino acid frequencies occurring in the substrate sequences as done by others^57^. An example of the use of the simulated background databases for normalization is illustrated in the following section.

### Potential pitfalls in data analysis: (ii) peptide product database size

The second factor in our list of potential pitfalls refers to the peptide product database size. To investigate the impact of the peptide product database size on the statistical analysis of PCPS features, we compared the amino acid frequency at sP_1_ and sP_1_’ sites between the peptide sequences originally published by Paes *et al*.^57^ and ProteasomeDB. In both peptide product databases, the obtained amino acid frequencies were normalized by the frequencies in the respective simulated background database (discussed in the section above). This was done to account for potential biases introduced both through natural variation of amino acid frequency and substrate composition (see above). Paes *et al*. compared the splice-site signature of forward *cis*- and reverse *cis*-spliced peptides based on 130 *cis*-spliced peptides included in their analysis of 2 h *in vitro* degradation of 23 synthetic substrates. They concluded that forward and reverse *cis*-PCPS had a different preference for amino acids in sP1 and sP1’^57^. Their analysis was based on 63 forward and 67 reverse *cis*-spliced peptides from 15 substrates in the original Paes’ peptide product database, since they did not identify *cis*-spliced peptides from 8 synthetic substrates^57^. The corresponding subset of the ProteasomeDB - restricted to 2/4 h *in vitro* degradation of 71 synthetic substrates with 20 S standard proteasomes - included 1,674 forward and 1,080 reverse *cis*-spliced peptide products.

We repeatedly sampled a subset of peptide sequences (*i.e*., applied 200 bootstrapping iterations on 80% of the data) and calculated the normalized amino acid frequency in each sampling iteration. In general, the 90% confidence interval of all bootstrap iterations results in an estimation of both the amino acid frequency at the sP1 and sP1’, and the robustness of this estimation. Accordingly, large confidence intervals indicate low reliability of the obtained amino acid frequencies.

The confidence intervals of the original Paes’ peptide product database^57^ were always larger than the ProteasomeDB subset (*e.g*., see A, C, H, Q amino acids in sP_1_’ of reverse *cis*-spliced peptides; Fig. 8b). For many amino acids, the original Paes’ peptide product database showed almost zero frequency at sP_1_ and sP_1_’, which may suggest that these amino acids were not used by proteasomes as splice-sites. This was not confirmed on the ProteasomeDB subset (*e.g*., see N, S, T, V amino acids in sP_1_’ of reverse *cis*-spliced peptides; Fig. 8b). At last, for most of the amino acids, the normalized frequency computed from the original Paes’ peptide product database^57^ and the ProteasomeDB subset did not match (Fig. 8b). All these analyses point toward the risk of overinterpretation of results obtained from small spliced peptide product databases, which may also explain the different results obtained in the three datasets shown in Figs. 7,8b.

From this preliminary analysis, we observed pools of amino acids that were either favored or disfavored as sP1 and sP1’, thereby suggesting that PCPS has peptide sequence preferences. Future studies, perhaps using grouping strategies based on chemophysical features of amino acids, could use ProteasomeDB to decipher the peptide sequence preferences of both peptide hydrolysis and peptide splicing catalyzed by 20 S proteasomes.

### Potential pitfalls in data analysis: (iii) synthesis errors

The third factor in our list of potential pitfalls refers to a confounding element in this type of sample, *i.e*. the presence of synthesis errors and their elimination. Both invitroSPI and invitroPB developed (different) strategies for synthesis error removal (Fig. S1, S2). Between the two methods, invitroSPI has likely the most stringent strategy to eliminate synthesis errors, which might have been assigned as spliced peptides. Indeed, it discards not only peptides identified as such in the control sample (either 0 h digestions or samples with synthetic substrates and no proteasomes) but also any putative spliced peptide with the same splice-site as a synthesis error identified in the control samples (see Figs. 1f, 3a, 4a). The latter step, which is not present in the invitroPB pipeline, may result in the elimination of spliced peptides that are produced by proteasomes although a peptide with the same splice-site is also present as synthesis error. In addition, both methods do not eliminate spliced peptides that have, as C-terminal splice-reactants, non-spliced peptides, which are present in the control samples and, thus, are assigned as synthesis errors. For example, we computed that a fraction of spliced peptides (median 6.4**%** per substrate) contained a potential non-spliced synthesis error as their C-terminal splice-reactant in ProteasomeDB. In the same peptide product database, the fraction of spliced peptides that contain a non-spliced peptide – that is not a synthesis error – as their C-terminal splice-reactant is 36.5%. The former circumstance might be considered for further analysis, depending on the user’s choice and analysis goal. Indeed, for these cases, no unequivocal statement about the origin of the C-terminal splice-reactant can be made (they could be generated as non-spliced peptides by proteasomes even if they are also present as synthesis errors), and, regardless of the splice-reactant’s origin, all these splice-reactants underwent PCPS to generate a splice-peptide. Therefore, the splice-reactants matched the catalytic requirements of PCPS in terms of sequence length and composition, and, hence, could be included in the downstream analysis depending on the analysis objective.

To this end, we have a feature in ProteasomeDB, which denotes whether a spliced peptide potentially contains a C-terminal splice-reactant that could be a synthesis error (**Tab. 1**). This information is limited to C-terminal splice-reactants because, according to the transpeptidation model of PCPS the N-terminal splice-reactant needs to be first cleaved by proteasomes to form the acyl-enzyme intermediate, and, thus, it cannot be a synthesis error. There are only few examples of PCPS via other reaction mechanisms such as condensation^20,25^.

### Potential pitfalls and missed opportunities in data analysis: (iv) restriction in peptide and splice-reactant length

The fourth factor in our list of potential pitfalls refers to the restriction of features of spliced and non-spliced peptides that can be identified. Both invitroSPI and invitroPB developed different strategies for non-spliced and spliced peptide identification, which can impinge upon the pool of identified peptide products. For example, Paes *et al*.^57^ restricted the identification to *cis*-spliced peptides longer than 7 amino acids, whereas non-spliced peptides had no restriction of this kind. This could explain why Paes *et al*.^57^ described a narrower length distribution for *cis*-spliced peptides than non-spliced peptides. In contrast, invitroSPI, which applies the same identification strategy to non-spliced and spliced peptides regarding their length restrictions (5 residues or longer; see Fig. 2), did not confirm the result of Paes *et al*.^57^. In fact, the analysis of the whole dataset using invitroSPI showed that proteasome-generated *cis*-spliced peptides, and *trans*-spliced peptides, are on average longer than non-spliced peptides in the ProteasomeDB (Fig. 7b), as previously shown in the Specht database of peptide products^8^.

InvitroPB also forbids the identification of spliced peptides with a splice-reactant length of one amino acid (Fig. 2). This strategy was in part based on a single example of a *cis*-spliced epitope previously described by Michaux *et al*.^51^ (*i.e*., gp100_195-202/192_). It has been demonstrated *in vitro* and *in cellula* that this specific *cis*-spliced epitope is not spliced as such, but as a C-terminal extended precursor, with a splice-reactant that is three amino acids long. However, upon PCPS, the spliced epitope precursor can be further processed by proteasomes, thereby generating the *cis*-spliced epitope that is recognized by CD8^+^ T cells of melanoma patients^20,51^. In contrast to invitroPB, invitroSPI identifies *cis*-spliced peptides with a one amino acid-long splice-reactant as final products (Fig. 2), since they could be the result of an initial PCPS event followed by peptide hydrolysis. In our analysis, these *cis*-spliced peptides had a comparable MS2 spectrum quality as the other identified peptides (Fig. 4g), thereby supporting their reliable identification.

It is also worth noting that in *in vitro* digestions of synthetic polypeptides with proteasomes, MS measurements and the downstream analysis identify the final products of the PCPS reaction rather than intermediate products, and, hence, we can never exclude that an identified peptide is the outcome of multiple peptide catalytic reactions. As a consequence, it is non-trivial to draw conclusions about the minimal length of splice-reactants in such datasets of proteasome-generated spliced peptides. Spliced peptides can re-bind to the proteasome’s active site and be cleaved, thus resulting in shorter splice-reactants than in the original transpeptidation reaction. By including spliced peptides with a splice-reactant length of one amino acid in ProteasomeDB, we could, however, carry out a simple analysis to understand if the pioneering observation of Michaux *et al*.^51^ could be generalized. To this end, we investigated the length of (i) N-terminal splice-reactants of forward *cis*-spliced peptides that originate from the substrate’s N-terminus and (ii) C-terminal splice-reactants of forward *cis*-spliced peptides that originate from the substrate’s C-terminus. The frequency of short fragments as splice-reactants that were located at the substrate’s termini could allow conclusions to be drawn about the minimal splice-reactant lengths required for PCPS since these splice-reactants could not be derived from trimming of longer fragments (Fig. S8a). In ProteasomeDB, we identified many forward *cis*-spliced peptides with N-terminal splice-reactants located at the substrate’s N-terminus, among which around 4.5% were one amino acid long and 9.2% were two amino acids long (Fig. S8b). This analysis suggested that N-terminal splice-reactants of one amino acid length could be efficiently used as such for PCPS. On the contrary, forward *cis*-spliced peptides with a one amino acid long C-terminal splice-reactant located at the substrate’s C-terminus were identified far less frequent (Fig. S8b), thereby suggesting that the C-terminal splice-reactants of at least 2 amino acids length were required for an efficient PCPS. Overall, this result confirmed the initial observation of Michaux *et al*.^51^, which was limited to C-terminal splice-reactants, although exceptions have been reported in ProteasomeDB. Furthermore, the relative frequency of spliced peptide products with a one amino acid long splice-reactant seemed to be smaller in 2/4 h vs 20/24 h digestion experiments (Fig. S8c). Similarly, the splice-reactant length distribution appeared narrower at later digestion time points compared to earlier time points, although not statistically significant (Fig. S8d). These data could be due a re-entry of spliced peptides followed by peptide hydrolysis in the late time point of the reactions, as well a change in proteasome dynamics over time as shown in other experimental set up^33^.

### Potential pitfalls and missed opportunities in data analysis: (v) restriction in peptide identification based on their location within the substrate

InvitroSPI and invitroPB differed in another aspect of the peptide product identification strategy, which impinged upon the features of the identified peptide pool. InvitroSPI allowed the identification of non-spliced and spliced peptides carrying N- or C-termini of synthetic polypeptide substrates. These peptides were also identified by invitroPB, which, however, excluded them in the downstream analysis.

To understand if their exclusion could bias the analysis of the identified peptide products, we computed *in silico* the theoretical fraction of non-spliced and *cis*-spliced peptides carrying the N- or C-termini of the substrates of the whole dataset, by applying the peptide length restrictions applied by invitroSPI and invitroPB. The fraction of theoretical peptides carrying the substrate’s N- or C-termini strongly depended on the substrate length (Fig. S9). This theoretically expected frequency was not confirmed among the fractions of experimentally identified peptides which carried the substrate’s N- or C-termini, analyzing the PB dataset and the whole dataset by applying the two identification methods (invitroSPI and invitroPB), respectively (Fig. S9 and Fig. 7a). We observed that while the fraction of identified non-spliced peptides that carry either of the substrate’s termini lay within the theoretically expected range, the fraction of spliced peptides with this property was much higher than expected by chance (Fig. S9). The similarity between measured and predicted MS2 spectra of spliced peptides with or without substrate’s N- or C-termini did not differ among the peptides identified in the PB dataset (Fig. 4g), hence suggesting that their identification was equally reliable. Therefore, by removing spliced and non-spliced peptides carrying the substrate’s N- or C-termini, one would not only remove a large portion of peptides produced by proteasomes *in vitro*, but also introduce a bias in the analysis by artificially constraining the spliced peptide pool. Furthermore, and in line with our observations, there is preliminary evidence of preferential processing of protein termini by proteasomes in living cells. Indeed, a larger frequency of non-spliced peptides produced by proteasomes by peptide hydrolysis of the termini of proteins compared to their central area has been shown *in cellula* by the pioneering work of Wolf-Levy and colleagues^71^. It would be worthwhile to verify in the same kind of samples, *i.e*., peptides eluted from proteasomes *in cellula*, if this holds also true for spliced peptides.

### Potential pitfalls in data analysis: (vi) non-spliced peptides with PTMs

Another example of a different strategy between invitroSPI and invitroPB, which could have an impact on the features of the identified peptide pool, is related to chemical PTMs. InvitroSPI allowed the identification of both non-spliced and spliced peptides carrying three chemical modifications (see ‘Technical aspects of invitroSPI’ chapter), and thus treated non-spliced and spliced peptides equally for this aspect. In contrast, invitroPB filtered out PTM-labelled non-spliced peptides, and introduced a specific filter only for *cis*-spliced peptides. We believe that the exclusion of PTM-labelled non-spliced peptides in the final list of identified peptides was a specific strategy adopted by Paes *et al*.^57^ for the comparison of non-spliced and *cis*-spliced peptides in that study, and, thus, could potentially be omitted in future applications of invitroPB. Conversely, PTMs had a key role in the identification of *cis*-spliced peptides by invitroPB: the method excluded MS2 spectra potentially assigned to *cis*-spliced peptides if they might have been non-spliced peptides tagged with any of the 313 PTMs considered by PEAKS-PTM. The original objective of Paes *et al*.^57^ was to reduce the risk of miss-assignment of MS2 spectra to *cis*-spliced peptides. This step of invitroPB, which was embedded in the method pipeline, may have achieved the original objective, although it may have also resulted in a reduced recall of *cis*-spliced peptides. As indicated in Fig. 3b and Fig. 4b,c, invitroSPI assigned several PSMs to spliced peptide sequences, which were dismissed by invitroPB because they were identified as non-spliced peptides with PTMs by PEAKS-PTM. The competition of different peptide sequences for the assignment of a MS2 spectrum in the presence of a large search space is an issue that has been addressed with various strategies^72,73^ and benchmarking approaches^44,74^. It is worth noting that the sequence search space of PEAKS-PTM, which considers 313 PTMs (maximum two PTMs allowed per peptide), may be even larger than the spliced peptide sequence search space, and thus be tangled to similar statistical issues. Therefore, the *a priori* exclusion of MS2 spectra for spliced peptide identification because they might be non-spliced peptides with unlikely PTMs (Fig. 3c) may not be directly supported by statistical considerations. In our opinion, any PTM-modified peptide assigned by PEAKS-PTM should be revisited to understand if it could occur in the specific experimental context. In the present study, technical modifications such as formylations could be explained through the use of formic acid in the MS buffer. On the contrary, biological modifications such as phosphorylation, ubiquitination and others, although suggested by PEAKS-PTM, are most likely false positive assignments (Fig. 3c). Nevertheless, an interesting avenue of further research could be to investigate to what extent PTMs occur before or after the splicing/hydrolysis reaction, and to what extent they influence the reaction towards either splicing or hydrolysis.

### Potential pitfalls in data analysis: (vii) multi-mapper peptides

The last issue that we would like to mention is the presence of peptide sequences that may have different locations within the substrate sequence, *i.e*., multi-mapper peptides. In invitroSPI, we imposed a hierarchical strategy, which gives preference to non-spliced over spliced peptides, and *cis*-spliced over *trans*-spliced peptides (see Methods section). Nonetheless, many *cis*-spliced peptide sequences may be both forward and reverse *cis*-spliced peptides; many spliced peptide sequences may be spliced peptides with different splice-reactant lengths, and hence different splice-sites. This issue has not been considered by previous studies on both *in vitro* digestions of synthetic polypeptides by proteasomes^8,57^, and HLA-I immunopeptidomes^10–14^. These studies adopted simple random assignment strategies, which may lead to artefacts. We think that more elaborated biochemical approaches should be used to better define the origin of these multi-mapper spliced peptides to avoid bias in the development of PCPS predictors. ProteasomeDB could be a cornerstone of such studies.

## Usage Notes

The whole peptide product database - ProteasomeDB - is provided as CSV file, which can be opened in Excel or any text editor.

## Supplementary information

SI

## Data Availability

The algorithm generating all possible *cis* and *trans* spliced peptides was originally described by Liepe *et al*.^63^. InvitroSPI method has been implemented with Snakemake in the Conda environment and is available at GitHub (https://github.com/QuantSysBio/invitroSPI). The analysis scripts (written in R) and implementation of invitroPB are available on Figshare online repository^70^. Analyses were carried out in R v4.1.1. Figures have been generated in R and postprocessing was done with Adobe Illustrator v25.2.3. The new *in vitro* TSN2 and TSN89 digestion samples were measured on Fusion Lumos Orbitrap, and acquired using Xcalibur v4.4.
