# Supplementary material for: InvitroSPI and a large database of proteasome-generated spliced and non-spliced peptides
Source: Sci Data. 2023 Jan 10;10:18. doi: 10.1038/s41597-022-01890-6 (PMC9832164; doi:10.1038/s41597-022-01890-6)
Supplement: Supplementary file 1 — SI [file 41597_2022_1890_MOESM1_ESM.pdf]

**InvitroSPI and a large database of proteasome-generated spliced and non-spliced peptides**

Correspondence to: Michele Mishto (michele.mishto@kcl.ac.uk) & Juliane Liepe (jliepe@mpinat.mpg.de).



| Item | Title | Page |
|---|---|---|
| Table S1 | Gp100-PMM_210325 synthetic peptide library | 2 |
| Figure S1 | InvitroSPI pipeline | 3 |
| Figure S2 | InvitroPB pipeline | 4 |
| Figure S3 | Theoretical peptide search space for spliced and non-spliced peptide products | 5 |
| Figure S4 | Estimated experimental FDRs for each peptide product type identified by applying invitroSPI with a delta score range on the PB dataset | 5 |
| Figure S5 | Peptide yield in the original study of Paes *et al.* and in invitroPB using two PEAKS versions | 6 |
| Figure S6 | Spectral angle distribution of measured and predicted MS2 spectra of non-spliced peptides is influenced by peptide length | 7 |
| Figure S7 | Sequence overlap between different peptide product databases | 8 |
| Figure S8 | Short splice-reactants at substrate termini in early and late time points of *in vitro* digestions | 9 |
| Figure S9 | Fraction of non-spliced and *cis*-spliced peptides carrying the N- or C-termini of synthetic polypeptide substrates depending on substrate length | 10 |

| **Peptide sequence** | **Position within TSN2 substrate sequence** |
|---|---|
| RTKQLYPEW | 6-8/13-18 |
| AWNR | 9-12 |
| QLYPEWTEAVSRQL | 13-21/1-5 |
| RQYPEWTEAQR | 3-4/15-23 |
| RQYPQWTEAQR | 3-4/15-16/4/18-23 |
| VSRQLVSRQL | 1-5/1-5 |
| VSRQLVSRQLRT | 1-5/1-7 |
| NRQLYPEWVSR | 11-18/1-3 |
| QLYPEWVSR | 13-18/1-3 |
| YPEWVSR | 15-18/1-3 |
| QLYPEWRTK | 13-18/6-8 |
| QLYPEWTTEAQR | 13-19/19-23 |
| YPEWVSRQL | 15-18/1-5 |
| QLYPEWTEARTKAW | 13-22/6-10 |
| QLYPEEWTEAQR | 13-17/17-23 |
| RTK | 6-8 |
| VSRQL | 1-5 |
| RTKAWNR | 6-13 |
| AWNRQLYPEW | 9-18 |
| QLYPEWRTKAWNR | 13-18/6-12 |
| RTKAWNRQL | 6-14 |
| NRQLYPEW | 11-18 |
| LYPEW | 14-18 |
| RTKA | 6-8 |
| YPEW | 15-18 |

**Table S1. Gp100-PMM_210325 synthetic peptide library**. The synthetic peptides present in the peptide library at a concentration of 0.4 μM (each peptide). They may derive from the 23 amino acid long synthetic polypeptide TSN2 [VSRQLRTKAWNRQLYPEWTEAQR] as either non-spliced, *cis*-spliced or *trans*-spliced peptides or as splice-reactants. The exception is the synthetic peptide [RQ][YP][Q][WTEAQR], which would need to be generated through multiple peptide splicing events, and represents here a "trapping peptide" for the method validation. Many of these peptides, although not all of them, were previously identified *in vitro* and, in some cases, also *in cellula* [1-6].

**invitroSPI**

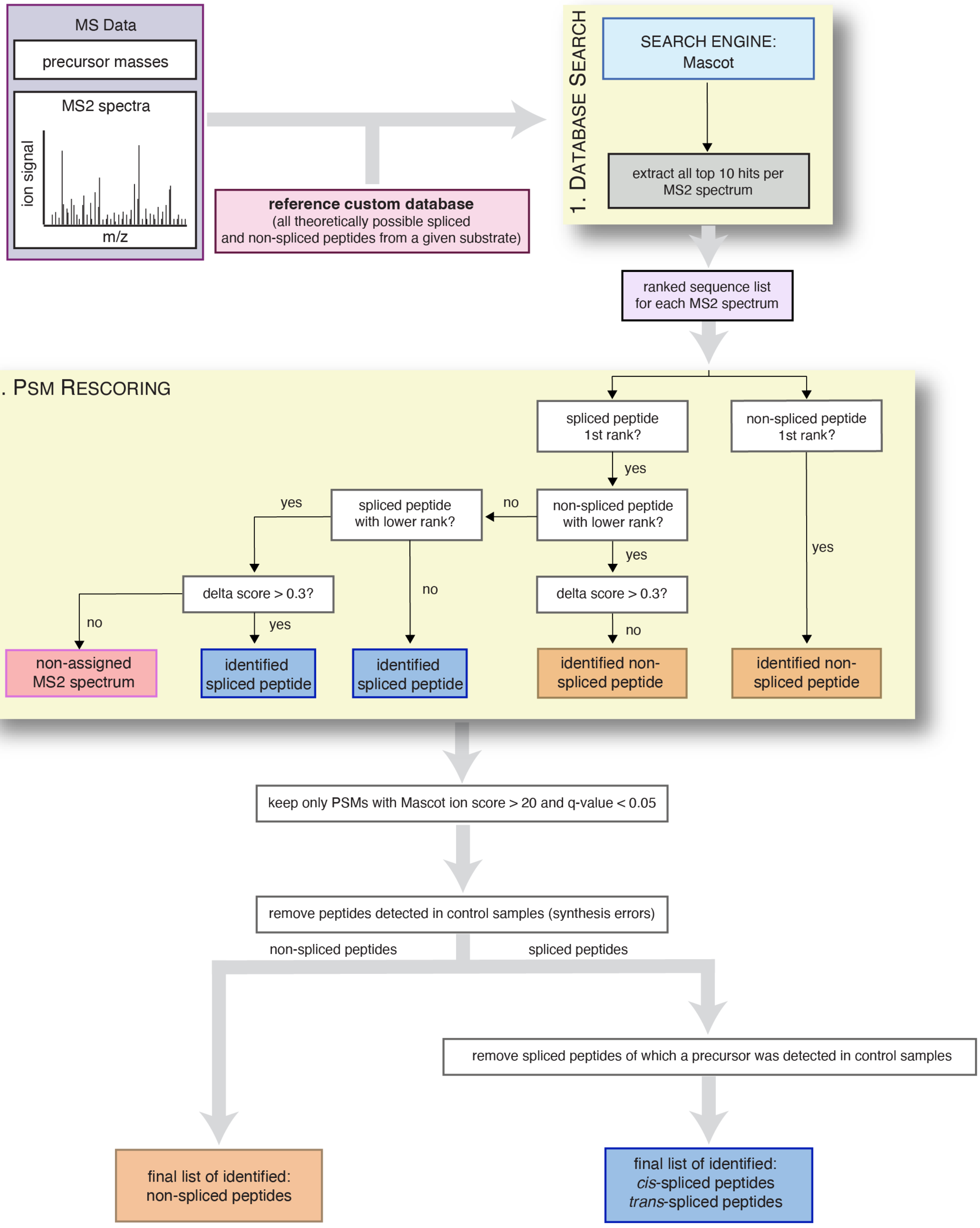


**Figure S1. InvitroSPI pipeline**.

**invitroPB**

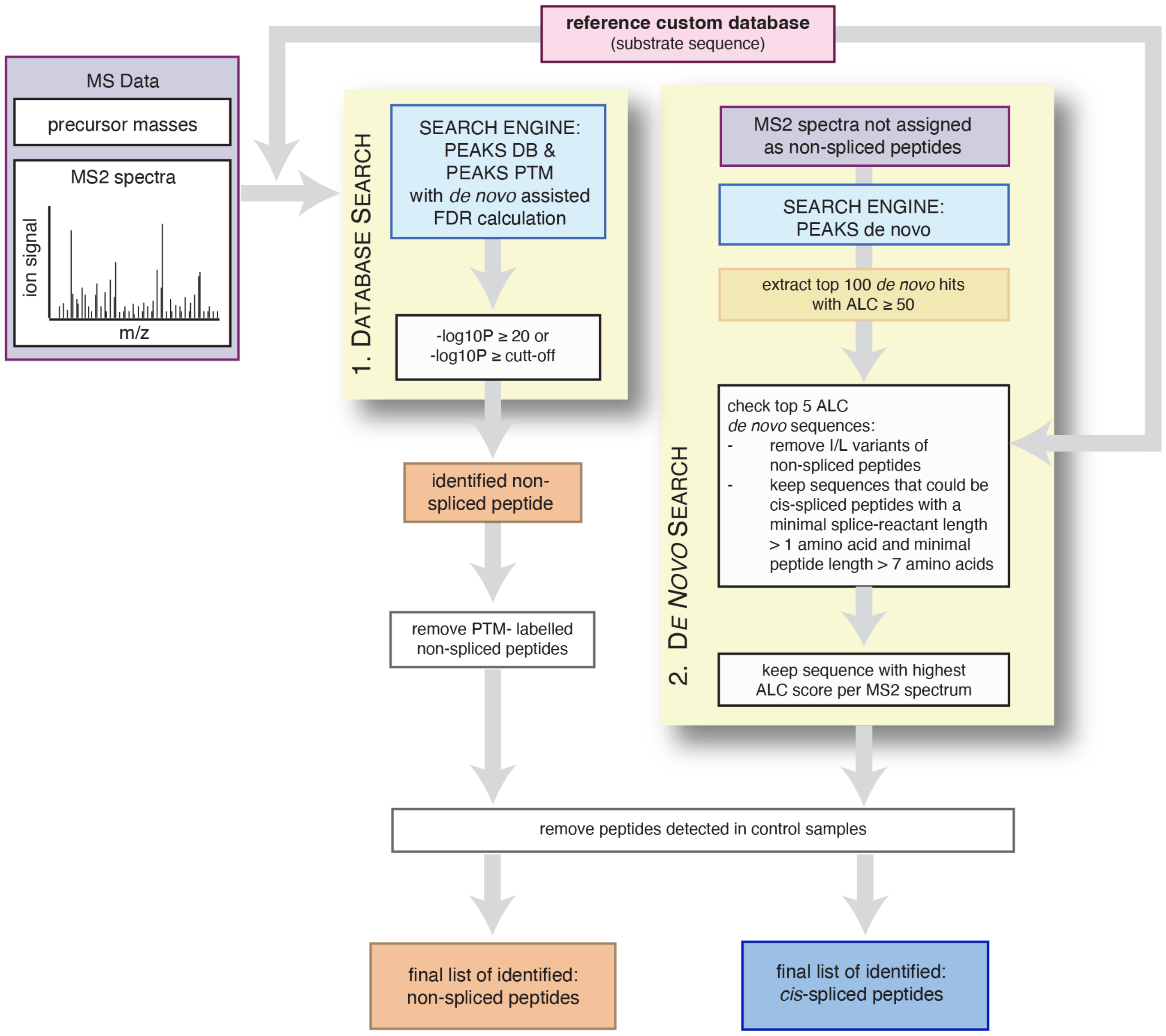


**Figure S2. InvitroPB pipeline.**

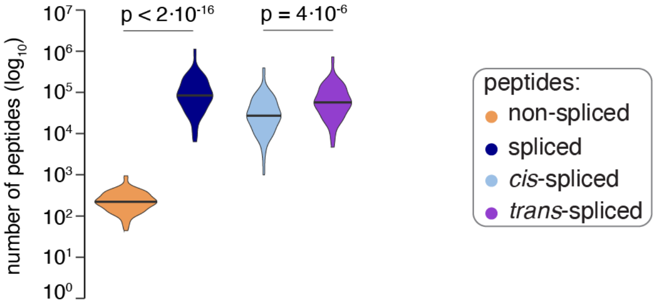


**Figure S3. Theoretical peptide search space for spliced and non-spliced peptide products.** Violin plots show the distribution of sequence numbers that could theoretically be derived from the synthetic polypeptide substrates in sequence-agnostic fashion for non-spliced, spliced, *cis*-spliced and *trans*-spliced peptides. They form the theoretical sequence search space of the whole dataset. Medians are indicated as horizontal lines. Calculations were carried out on the substrate sequences (n = 80) in the whole dataset that were digested with 20S standard proteasomes. The theoretical search space differs significantly between spliced and non-spliced peptides and between *cis*- and *trans*-spliced peptides. Significant p values of a two-samples Wilcoxon test are reported.

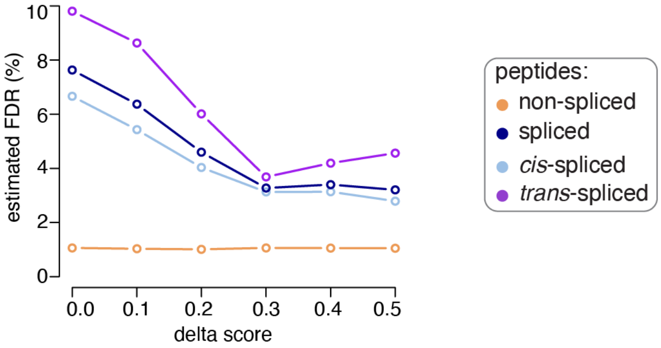


**Figure S4. Estimated experimental FDRs for each peptide product type identified by applying invitroSPI with a delta score range on the PB dataset.** Estimation of the FDRs of non-spliced, *cis*-spliced, *trans*-spliced, and all spliced peptides identified by applying invitroSPI to PB dataset (*in vitro* digestions of 25 synthetic substrates with proteasomes). FDRs of spliced peptide identifications were estimated at 1 % FDR of non-spliced peptides, based on the spectral angle distribution between normalized, measured and predicted MS2 spectra of non-spliced, *cis*-spliced, and *trans*-spliced peptides identified by applying invitroSPI with a delta score range. MS2 spectra were predicted by applying Prosit algorithm [7].

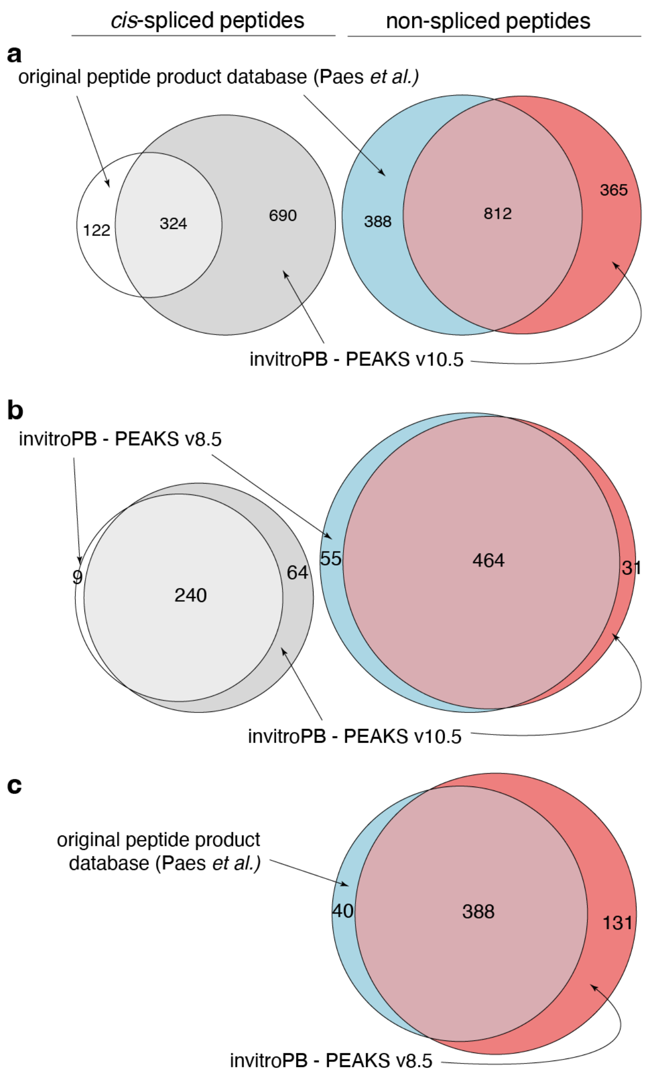


**Figure S5. Peptide yield in the original study of Paes *et al.* and in invitroPB using two PEAKS versions. a**) Overlap between non-spliced and *cis*-spliced peptides either described in Paes *et al.* [8] or identified by invitroPB using PEAKS v10.5 on TSN100-124 substrates (the PB dataset). **b**) Overlap between non-spliced and *cis*-spliced peptides identified by invitroPB using either PEAKS v8.5 or PEAKS v10.5 on TSN100-102 substrates (the short substrates in the PB dataset). **c**) Overlap between non-spliced peptides described either in Paes *et al.* [8] or identified by invitroPB using PEAKS v8.5 on TSN122-124 substrates (the long substrates in the PB dataset). In (**a-c**) unique peptides, which were identified in the 2h *in vitro* digestions of the synthetic substrates. Non-spliced and spliced peptides that contained the N- or C-termini of the substrates were included in this analysis, since they were reported in the original peptide product database of Paes *et al.* [8]. Paes *et al.* published a *corrigendum* of the original peptide product database in 2021 [9].

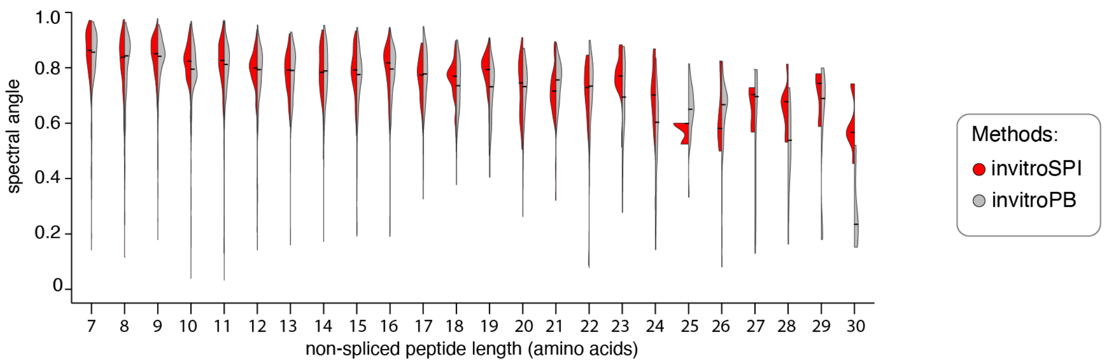


**Figure S6**. **Spectral angle distribution of measured and predicted MS2 spectra of non-spliced peptides is influenced by peptide length**. Spectral angle distribution between normalized, measured and predicted MS2 spectra of non-spliced peptides identified by applying either invitroSPI or invitroPB to PB dataset. In the violin plots, horizontal black lines represent the median. Prediction of MS2 spectra was carried out by applying Prosit algorithm version 2020 [7]. In the violin plots, horizontal black lines represent the median. The length of the peptide products here analyzed is limited to 7 amino acid residues or longer, despite invitroSPI detected also shorter peptides, because Prosit was only trained on 7 amino acid long peptides or longer [10].

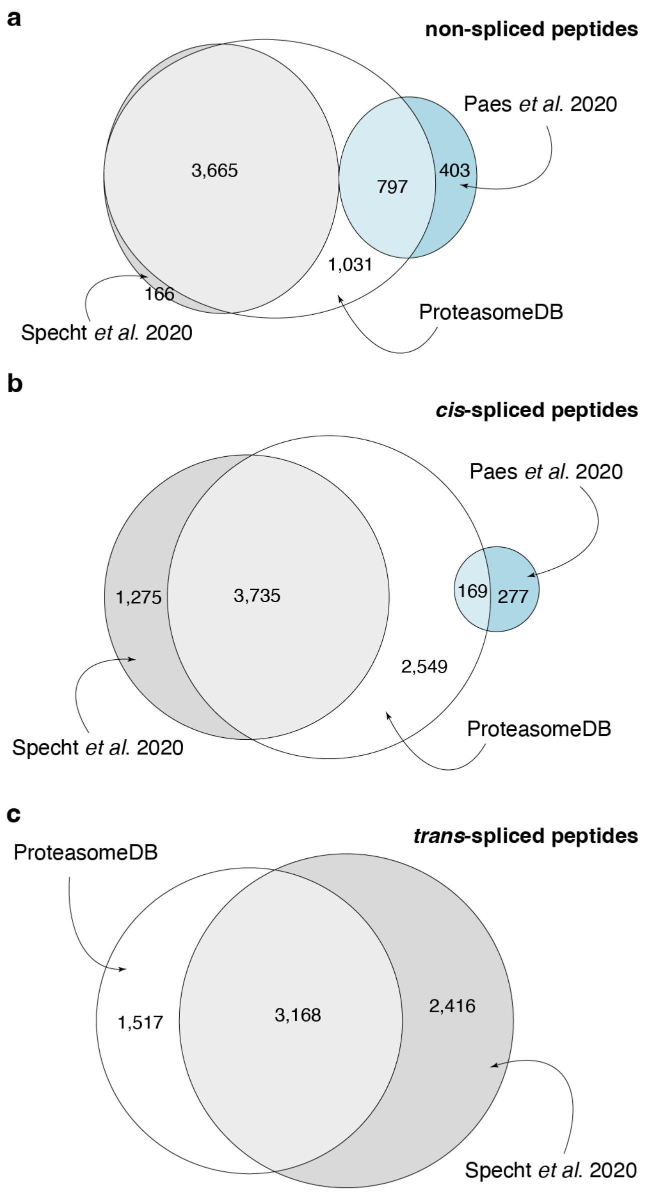


**Figure S7. Sequence overlap between different peptide product databases. a-c**) Overlap of (**a**) non-spliced, (**b**) *cis*-spliced and (**c**) *trans*-spliced peptide sequences in ProteasomeDB, as well as the sequences originally published by Specht *et al.* [3] and Paes *et al.* [8]. The number of unique peptides per substrate is here reported.

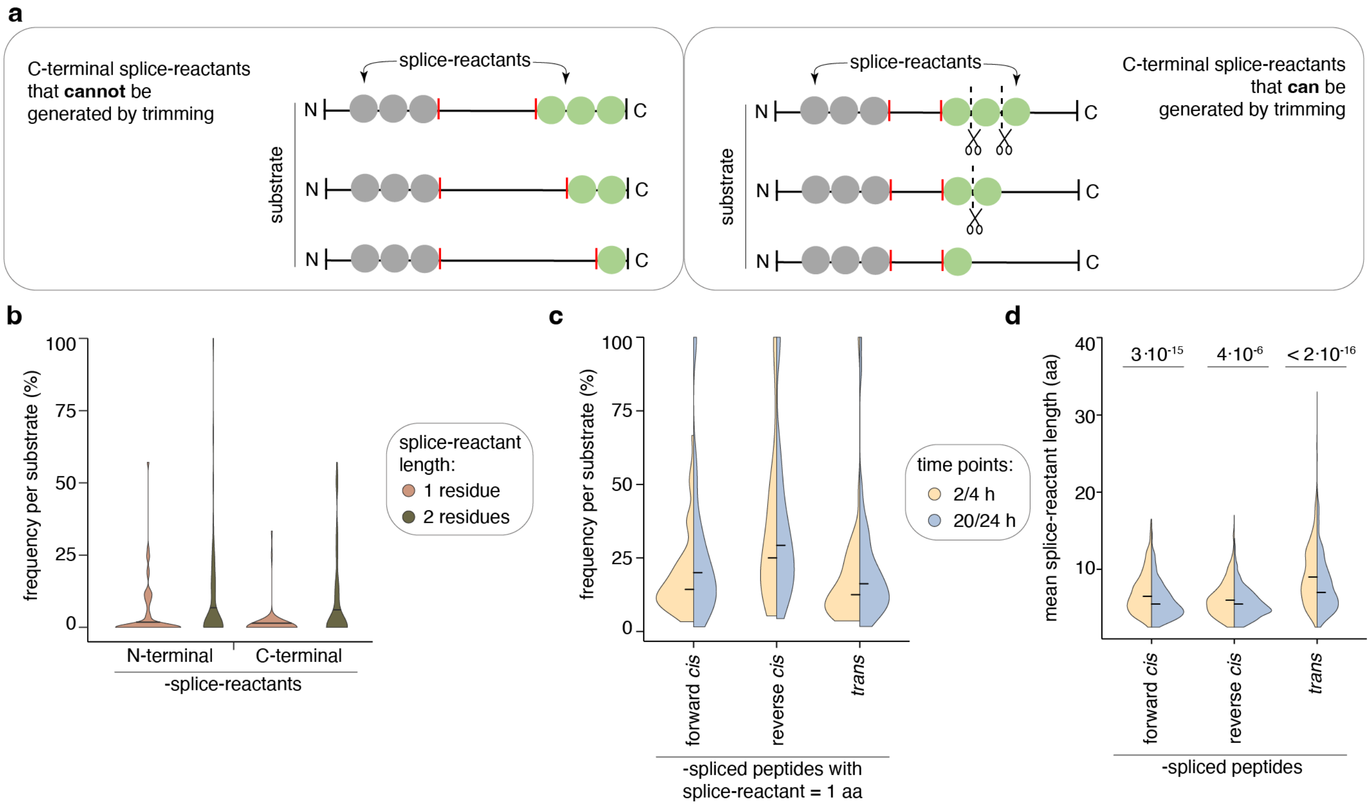


**Figure S8. Short splice-reactants at substrate termini in early and late time points of *in vitro* digestions.** (**a**) Schematic of minimal splice-reactant length analysis. C-terminal splice-reactants of forward *cis*-spliced peptides that originate from the substrate's C-terminus can only be generated without trimming after the transpeptidation reaction. On the contrary, C-terminal splice-reactants of forward *cis*-spliced peptides that do not originate from the substrate's C-terminus can also be generated via C-terminal trimming after the transpeptidation reaction. Circles denote amino acids. Red bars denote the splice-site. (**b**) Shown are the frequencies of forward *cis*-spliced peptides located at the substrate's N-terminus, whose N-terminal splice-reactant is one or two amino acids long, respectively, compared to all forward *cis*-spliced peptides located at the substrate's N-terminus. Similarly, the frequencies of forward *cis*-spliced peptides located at the substrate's C-terminus, whose C-terminal splice-reactant is one or two amino acids long, respectively, compared to all forward *cis*-spliced peptides located at the substrate's C-terminus, is shown in the right side of the chart. (**c**) Frequency of spliced peptides containing a one or two amino acid long splice-reactant (not necessarily at the substrate's termini) at early (2/4 h) and late (20-24 h) time points *in vitro* digestions. (**d**) Distribution of mean splice-reactant lengths in spliced peptide products identified at early and late time points in *in vitro* digestions. In the violin plots, horizontal black lines represent the median. In (**b-d**), the spliced peptides are those identified by applying invitroSPI to *in vitro* digestions of the synthetic polypeptides TSN100, TSN101, TSN102, TSN103, TSN104, TSN105, TSN106, TSN107, TSN108, TSN109, TSN110, TSN111, TSN112, TSN113, TSN115, TSN116, TSN117, TSN118, TSN119, TSN120, TSN121, TSN122, TSN123, TSN124, TSN18, TSN2, TSN21, TSN22, TSN23, TSN3, TSN41, TSN42, TSN43, TSN44, TSN45, TSN46, TSN49, TSN50, TSN52, TSN53, TSN54, TSN55, TSN56, TSN57, TSN58, TSN59, TSN60, TSN61, TSN62, TSN63, TSN64, TSN65, TSN66, TSN69, TSN7, TSN70, TSN76, TSN77, TSN78, TSN79, TSN80, TSN81, TSN82, TSN83, TSN84, TSN85, TSN89, TSN90, TSN93. These are all synthetic substrates in the whole dataset that have both early and late time points measurements and were digested by 20S standard proteasomes. Statistically significant differences between early and late time points within the same type of spliced peptide product are reported. Only Kolmogorov-Smirnov test's p-values < 0.05 are reported and were considered significant.

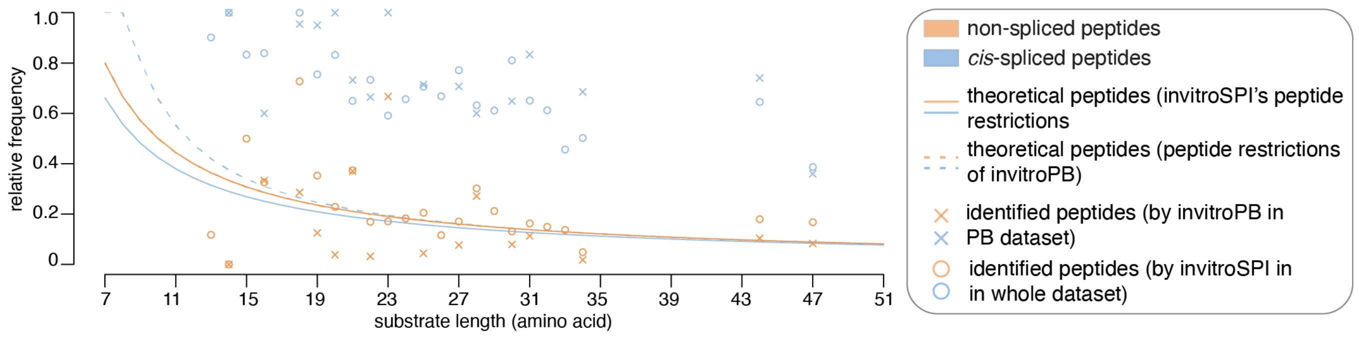


**Figure S9. Fraction of non-spliced and *cis*-spliced peptides carrying the N- or C-termini of synthetic polypeptide substrates depending on substrate length.** Lines show the *in silico* computation of the fraction of theoretically possible peptide products containing the substrate's N- or C-termini, depending on substrate length (in amino acids), and considering the peptide length restrictions of invitroSPI and invitroPB. For invitroSPI (solid lines), restrictions are a minimal splice-reactant length of one amino acid, and a minimal peptide length of five amino acids. For invitroPB (dashed lines), restrictions are a *cis*-spliced peptide's minimal length of eight amino acids and a minimal splice-reactant length to two amino acids, as well as a minimal non-spliced peptide length of five amino acids. Dots and crosses represent the fraction of peptide products which carry the substrate's N- or C-termini, identified either through invitroPB in PB dataset (crosses) or through invitroSPI in the whole dataset (dots). Values refer to unique peptides from all time points digested with all proteasome isoforms.

The squared difference between theoretically possible and identified fractions of products carrying the substrate's N- or C-terminus in the whole dataset analyzed using invitroSPI have been tested against the hypothesis of zero difference using a two-sided Student's t-test (against a normal distribution). The discrepancy between theoretically possible and identified fractions is significant for *cis*-spliced peptides ($p = 6.7*10^{-11}$) and non-spliced peptides ($p = 0.0437$).

**Reference**

1 Ebstein, F. *et al.* Proteasomes generate spliced epitopes by two different mechanisms and as efficiently as non-spliced epitopes. *Sci Rep* **6**, 24032 (2016).
2 Mishto, M. *et al.* Driving Forces of Proteasome-catalyzed Peptide Splicing in Yeast and Humans. *Mol Cell Proteomics* **11**, 1008-1023 (2012).
3 Specht, G. *et al.* Large database for the analysis and prediction of spliced and non-spliced peptide generation by proteasomes. *Sci Data* **7**, 146, doi:10.1038/s41597-020-0487-6 (2020).
4 Dalet, A., Stroobant, V., Vigneron, N. & Van den Eynde, B. J. Differences in the production of spliced antigenic peptides by the standard proteasome and the immunoproteasome. *Eur J Immunol* **41**, 39-46 (2011).
5 Dalet, A., Vigneron, N., Stroobant, V., Hanada, K. & Van den Eynde, B. J. Splicing of distant Peptide fragments occurs in the proteasome by transpeptidation and produces the spliced antigenic peptide derived from fibroblast growth factor-5. *J Immunol* **184**, 3016-3024 (2010).
6 Vigneron, N. *et al.* An antigenic peptide produced by peptide splicing in the proteasome. *Science* **304**, 587-590 (2004).
7 Wilhelm, M. *et al.* Deep learning boosts sensitivity of mass spectrometry-based immunopeptidomics. *Nat Commun* **12**, 3346, doi:10.1038/s41467-021-23713-9 (2021).
8 Paes, W. *et al.* Elucidation of the Signatures of Proteasome-Catalyzed Peptide Splicing. *Front Immunol* **11**, 563800, doi:10.3389/fimmu.2020.563800 (2020).
9 Paes, W. *et al.* Corrigendum: Elucidation of the Signatures of Proteasome-Catalysed Peptide Splicing. *Front Immunol* **12**, 755002, doi:10.3389/fimmu.2021.755002 (2021).
10 Gessulat, S. *et al.* Prosit: proteome-wide prediction of peptide tandem mass spectra by deep learning. *Nat Methods* **16**, 509-518, doi:10.1038/s41592-019-0426-7 (2019).
